# Clinical and Treatment Characteristics of 3795 Adults Consecutively Hospitalized for Major Depressive Disorder in the OASIS-D Study

**DOI:** 10.1155/da/4470169

**Published:** 2025-11-26

**Authors:** Viktor B. Nöhles, Felix Bermpohl, Nikola Schoofs, Rouven Bathe-Peters, Nina Hilpert, Forugh Salimi Dafsari, Christine Reif-Leonhard, Andreas Reif, Susanne Schillo, Rabia Sana Arshad, Patricia Getty, Peter Falkai, Cornelius Schüle, Elena Wang, Mazda Adli, René Papenfuß, Andreas Meyer-Lindenberg, Stefan Fritze, Christian Otte, Dominique Piber, Livia Graumann, Sebastian Weyn-Banningh, Michael Bauer, Ute Lewitzka, Maik Spreer, Julia Clemens, Kerstin Rubarth, Christoph U. Correll

**Affiliations:** ^1^Department of Child and Adolescent Psychiatry, Psychosomatic Medicine and Psychotherapy, Campus Virchow-Klinikum, Charité – Universitätsmedizin Berlin, Corporate Member of Freie Universität Berlin and Humboldt-Universität zu Berlin, Berlin, Germany; ^2^German Center for Mental Health (DZPG), Partner Site, Berlin, Germany; ^3^Department of Psychiatry and Neurosciences, Campus Charité Mitte, Charité – Universitätsmedizin Berlin, Corporate Member of Freie Universität Berlin and Humboldt-Universität zu Berlin, Berlin, Germany; ^4^Department of Psychiatry and Psychotherapy, Faculty of Medicine and University Hospital Cologne, University of Cologne, Cologne, Germany; ^5^Department of Psychiatry, Psychosomatic Medicine and Psychotherapy, University Hospital Frankfurt – Goethe University, Frankfurt am Main, Germany; ^6^Fraunhofer Institute for Translational Medicine and Pharmacology ITMP, Frankfurt am Main, Germany; ^7^Clinic for Psychiatry and Psychotherapy, Ludwig-Maximilians-University Munich, Munich, Germany; ^8^German Center for Mental Health (DZPG), Partner Site, Munich, Germany; ^9^Max Planck Institute for Psychiatry, Munich, Germany; ^10^Department of Psychiatry and Psychotherapy, Campus Charité Mitte, Charité – Universitätsmedizin Berlin, Corporate Member of Freie Universität Berlin and Humboldt-Universität zu Berlin, Berlin, Germany; ^11^Center for Psychiatry, Psychotherapy and Psychosomatic Medicine, Fliedner Klinik Berlin, Berlin, Germany; ^12^Department of Psychiatry and Psychotherapy, Central Institute of Mental Health, Mannheim, Germany; ^13^German Center for Mental Health (DZPG), Partner Site, Mannheim, Germany; ^14^Department of Psychiatry and Psychotherapy, Campus Benjamin Franklin, Charité – Universitätsmedizin Berlin, Corporate Member of Freie Universität Berlin and Humboldt-Universität zu Berlin, Berlin, Germany; ^15^Department of Psychiatry and Psychotherapy, Faculty of Medicine, University Hospital Carl Gustav Carus, Technische Universität Dresden, Dresden, Germany; ^16^Institute of Biometry and Clinical Epidemiology, Charité – Universitätsmedizin Berlin, Corporate Member of Freie Universität Berlin and Humboldt-Universität zu Berlin, Berlin, Germany; ^17^Psychiatry Research, The Zucker Hillside Hospital, Northwell Health, Glen Oaks, New York, USA; ^18^Department of Psychiatry and Molecular Medicine, Zucker School of Medicine at Hofstra/Northwell, Hempstead, New York, USA

## Abstract

**Background:**

Major depressive disorder (MDD) is common and associated with high social and economic burden. Knowledge of characteristics of hospitalized adults with MDD can help identify clinical treatment and prevention targets.

**Methods:**

The multicenter “Patient Characteristics, Validity of Clinical Diagnoses and Outcomes Associated with Suicidality in Inpatients with Symptoms of Depression” (OASIS-D) study assessed characteristics of patients aged 18–75 years hospitalized between October 2020 and December 2024, who were admitted to a psychiatric inpatient unit for MDD at eight German centers. Baseline illness-, treatment-, and suicidality-related characteristics of the overall sample are reported.

**Results:**

Among 3795 patients (median age = 42.0, interquartile range [IQR] = 27.5–57.0 years; females = 53.9%) with MDD (severe episode = 75.3%, psychotic features = 7.9%; first episode = 34.9%; treatment-resistant depression [TRD] = 18.2%). Psychiatric comorbidities of MDD were present in 46.2% and included substance use disorder (18.9%), personality disorders (8.4%), stress/adjustment disorders (7.6%), and phobic/other anxiety disorders (6.6%). In 42.5%, the admission was prompted by a psychiatric emergency, primarily due to suicidality (35.0%), followed by stupor/refusal/intoxication/acute agitation (0.9%–1.5%), or danger to others/delirium (0.1%–0.3%). Overall, 72.0% of patients had active or passive suicidal thoughts, and 11.5% had attempted suicide within 2 weeks prior to admission. Furthermore, 83.9% had lifetime suicidal thoughts, and 36.0% had lifetime suicide attempts. Altogether, 76.8% had received outpatient psychiatric care within their lifetime (62.3% within 6 months), and 57.8% of patients had lifetime inpatient treatment for MDD. At admission, 71.6% of patients were prescribed psychiatric medications: antidepressants = 59.8%; antipsychotic = 25.1%, anxiolytics/hypnotics = 11.8%, and mood stabilizers = 8.6%. Additionally, 4.0% had previously received electroconvulsive therapy (ECT). The median hospitalization duration was 31.0 (IQR = 13.0, 57.0) days.

**Conclusion:**

Almost half of admissions in adults with MDD were considered emergencies, with 90% being related to suicidality, and only <60% received antidepressants at admission. These data underscore the need for early identification and treatment of adults with MDD, especially those with suicidality. Outcomes of this population required further study.

**Trial Registration:** ClinicalTrials.gov identifier: NCT04404309

## 1. Introduction

Major depressive disorder (MDD) is one of the most prevalent mental disorders globally and has been on the rise in recent decades [1]. The lifetime prevalence of MDD exceeds 10% [2, 3]. Individuals with MDD often exhibit significant functional impairment, including elevated rates of sick leave, diminished work productivity, and impaired daily activity [4]. Additionally, these individuals frequently experience substantial deficits in quality of life (QOL) across physical, psychological, and social domains, with many continuing to report markedly impaired QOL even after achieving remission [5, 6]. MDD is also associated with a high prevalence of passive and active suicidal thoughts and consequently a lifetime suicide attempt rate of over 30% [7–12]. The economic burden of MDD is substantial, encompassing both direct and indirect costs. Indirect costs include lost productivity, reduced work performance, placing an additional burden on individuals and society; direct costs are predominantly from inpatient treatment [13–15].

According to the German national practice guideline for MDD, inpatient treatment is strongly recommended for emergency cases, such as acute self- or other-directed harm or depressive stupor. Admission should be carefully evaluated when medical care is needed after a suicide attempt, when ongoing suicidality cannot be reliably assessed, or when a stable therapeutic relationship cannot be established despite initial treatment and the individual remains acutely suicidal. Further situations requiring evaluation for potential hospitalization include treatment resistance, risk of chronicity, severe agitation, pronounced psychotic symptoms, or impending depression-related stupor, self-neglect. Admission may also be necessary for comorbid substance use, decompensated somatic illnesses, or life circumstances severely hindering treatment success (e.g., requiring a change of environment) [16]. Given the critical nature of inpatient treatment, understanding the demographic and clinical characteristics of inpatients with MDD can assist in attempts to optimize care and reduce treatment costs.

Demographic and illness characteristics of inpatients with MDD are characterized by higher percentages of females, with means ranging from 53.1% to 73.0% [17–29]. The reported mean age of patients with MDD undergoing inpatient treatment, as observed across studies, ranged between 34.4 and 52.0 years [17, 22–28, 30, 31]. Most hospitalized patients with MDD suffer from severe depression without psychotic symptoms (39.6%–59.2%) [17, 20, 23, 32], followed by moderate depression (28.1%–48.8%) [17, 20, 23, 32], and severe depression with psychotic symptoms (11.6%–16.6%) [17, 20, 23, 32]. Across several inpatient samples, 51.0%–83.9% of patients were diagnosed with a single episode of MDD and 16.1%–49.0% with recurrent MDD [17, 26, 27, 33–36]. Treatment-resistant depression (TRD) has been described in 14.6%–29.3% of inpatients with MDD [24, 32]. Furthermore, at least one comorbidity was reported in 23.8%–42.5% [37–39] of cases, including anxiety/phobic disorders (12.8%–56.9%) [23, 25, 27, 29, 30, 34, 40–42], personality disorders (2.6%–23.1%) [17, 23, 24, 27, 29, 39, 41, 42], alcohol use disorder (5.0%–22.9%) [23–25, 28, 30, 34, 42], other substance use disorders (1.8%–17.6%) [23, 28, 30, 34, 42], and posttraumatic-stress disorder (PTSD; 3.7%) [43].

A Danish nationwide register study revealed that 50.2% [44] of hospitalized patients with MDD were classified as having psychiatric emergencies. Additionally, suicidality is highly associated with MDD, with frequencies in inpatients for suicide ideation ranging between 20.9% and 63.0% [22, 24, 25, 30, 36, 45, 46] for the time before or at admission and 67.0%–84.3% [47, 48] lifetime. Furthermore, suicide attempts ranged between 3.7% and 12.1% [24, 25] for the time before admission and 15.4%–57.8% [23, 26, 45, 46, 48] lifetime.

In a German naturalistic study of 193 inpatients with MDD, the referral context for admissions included 55.5% from a psychiatrist (private practice), 20.4% from a hospital transfer, 11.5% from an outpatient service, 4.7% from a psychotherapist (private practice), and 4.2% from a general practitioner (private practice) [36].

In prior studies, upon admission, a high proportion of patients with MDD received psychotropic medications, with a range of 76.7%–83.2% [30, 49] and a mean of 2.5 [32] in patients prescribed psychotropic medication. Among these patients, 40.2%–78.1% [22, 26, 28, 30] received antidepressants, including 25.0%–49.0% [26, 28, 49, 50] consisting of selective serotonin reuptake inhibitors (SSRIs), 17.4%–34.5% [26, 28, 49, 50] serotonin–norepinephrine reuptake inhibitors (SNRIs), and 2.1%–26.2% [26, 28, 34] tricyclic antidepressants (TCAs). Furthermore, during inpatient stay, upto 78.6% [30] of patients were prescribed an anxiolytic/hypnotic, 36.4%–60.1% [26, 28, 30, 34] antipsychotic medication, and 4.9%–16.5% [26, 28, 30, 34] mood stabilizers.

According to the literature, prior to inpatient care, 77.8% [42] of patients with MDD underwent outpatient psychotherapeutic treatment. Moreover, 5.5%–22.5% [23, 51–54] of hospitalized patients with MDD had received electroconvulsive therapy (ECT) during their lifetime. Finally, across multiple studies and healthcare systems, the average length of inpatient treatment in patients hospitalized for MDD ranged from 8.8 to 70 days [20, 26, 27, 33, 38, 55–57].

Identifying patient, illness, and treatment characteristics can improve the understanding of hospitalized patients with MDD. Moreover, this detailed information can guide the development of targeted interventions and service allocations, ensuring that treatment approaches are better aligned with the specific needs of the patients, thus enhancing overall treatment efficacy and patient care. Therefore, the objective of this study was to systematically characterize patients consecutively admitted to psychiatric inpatient units with a unipolar depressive episode. Based on the existing literature, we expected that the inpatient sample would be predominantly female, with severe or moderate MDD, that the admission would frequently be due to a psychiatric emergency consisting predominantly of suicidality-related events, and that lifetime suicidality and suicide attempts would be substantial, with a relevant minority of admissions following an acute suicide attempt. We further hypothesized that anxiety disorders, stress- and adjustment-related disorders, personality disorders, and substance use disorders would be frequent comorbidities, and that antidepressants would be the predominant psychiatric medication class prescribed upon admission.

## 2. Materials and Methods

### 2.1. Observational Design

This current study used baseline data from the multicenter “Patient Characteristics, Validity of Clinical Diagnoses and Outcomes Associated with Suicidality in Inpatients with Symptoms of Depression” (OASIS-D) study [58], conducted between October 2020 and December 2024, which assessed clinical characteristics in adults aged 18–75 years who were admitted consecutively to a psychiatric unit due to a single or recurrent episode of MDD (ICD-10 [59]: F32, F33) and retrospectively evaluated via chart review (for a detailed description of the study design, see [60]). OASIS-D was conducted between October 2020 and December 2024 at the following eight sites: three centers at the Charité–Universitätsmedizin Berlin, that is, (i) St. Hedwig Hospital, Department of Psychiatry and Psychotherapy (Charité–St. Hedwig), (ii) Charité Campus Mitte, Department of Psychiatry and Psychotherapy (Charité–Mitte), and (iii) Charité Campus Benjamin Franklin, Department of Psychiatry and Psychotherapy (Charité–CBF), (iv) Clinic for Psychiatry and Psychotherapy at the Ludwig-Maximilians-University Munich (LMU), (v) Department of Psychiatry, Psychosomatic Medicine and Psychotherapy at the University Hospital Frankfurt (KGU), (vi) Department of Psychiatry and Psychotherapy at the University Hospital of Cologne (Uni Köln), (vii) Department of Psychiatry and Psychotherapy at the University Hospital Carl Gustav Carus Dresden (UKD), as well as (viii) Central Institute of Mental Health, Department of Psychiatry and Psychotherapy in Mannheim (ZI Mannheim).

### 2.2. Participants

#### 2.2.1. Patient Inclusion Criteria

The study included both male and female patients aged between 18 and 75 years, who were admitted to a psychiatric inpatient unit and who had an ICD-10-based patient chart diagnosis of either a single depressive episode (ICD-10: F32) or a recurrent depressive episode (ICD-10: F33).

#### 2.2.2. Patient Exclusion Criteria

Excluded were psychiatric inpatients younger than 18 years and older than 75 years, those without a clinical diagnosis of a unipolar depressive episode, and those with a depressive episode in the context of bipolar disorder.

#### 2.2.3. Interventions and Assessments

Interventions prescribed at baseline were naturalistic based on pre-admission care. Similarly, the clinical-, illness-, and treatment-related characteristics were documented as part of usual care by the treating clinician, who in German hospitals is typically a resident physician or trained psychiatrist, each under the supervision of a senior attending. Data were subsequently extracted from the patient files by a study staff member and, in case of uncertainty, cross-checked with the treating clinicians. For data collection in the OASIS-D study, a structured Excel data collection form was used. Data were collected and sent to Charité in Excel Sheets in aggregated form from LMU, KGU, Uni Köln, UKD, and ZI Mannheim. Other study centers provided the data on an individual level via electronic case report forms (eCRFs). Missing data were identified and queried, and if possible missing data were added.

Collected data included demographic characteristics, specific MDD diagnoses, psychiatric comorbidities, suicidality characteristics, presence of TRD (two courses of antidepressant therapy with adequate dosages and an insufficient therapeutic effect), treatment prior to inpatient admission, inpatient treatment, and pharmacological treatment at admission. MDD diagnoses were categorized as mild (ICD-10: F32.0, F33.0), moderate (ICD-10: F32.1, F33.1), severe without psychosis (ICD-10: F32.2, F33.2), and severe with psychosis (ICD-10: F32.3, F33.3). Additionally, MDD diagnoses were classified as single episode (ICD-10: F32.x) or recurrent episode (ICD-10: F33.x). Suicidality was assessed by the treating clinician using the commonly employed definition in Germany as the presence of thoughts, actions, or intentions directed toward ending one's own life. Non-suicidal self-injurious behavior (e.g., cutting) is not included in this definition, and was therefore, not coded as suicidality. In terms of comorbid disorders, schizotypal and delusional disorders were categorized under schizoid personality disorder (see Table S1 for details of the F-diagnosis of comorbid psychiatric disorders). Regarding psychiatric medications, the mean and the number of medications overall and by type were provided by the Uni Köln only for patients starting from patient number 114 due to an administrative error; with complete recording at all other sites.

#### 2.2.4. Data Analysis

Descriptive statistics were utilized to characterize the sample, encompassing means and standard deviations for normally distributed numerical variables and medians with interquartile range (IQR) for non-normally distributed data, and frequencies and percentages for categorical variables. Percentages were calculated for different patient subgroups. Statistical analyses were performed using the software R-studio.

#### 2.2.5. Ethical Considerations

All methods were in accordance with relevant guidelines and regulations, including the Declaration of Helsinki, as well as additional clinical, federal state, and national guidelines. Prior to initiation of the study, the protocol received approval from the Ethics Committees of the Charité–Universitätsmedizin Berlin acting as the coordinating center (EA2/061/20) and from the Ethics Committees of the eight recruitment centers. For the current part 1 of the OASIS-D study and the baseline population, the study was exempt for patient informed consent, as this part was conducted as a retrospective patient chart review. For patients entering part 2 and 3 of the OASIS-D study that will be the focus of additional publications, informed consent was required and signed.

## 3. Results

### 3.1. Patient Characteristics

Overall, data from 3795 patients (Charité–St. Hedwig, *n* = 817; Uni Köln, *n* = 665; KGU, *n* = 607; LMU, *n* = 526; Charité–Mitte, *n* = 405; ZI Mannheim, *n* = 270; Charité–CBF, *n* = 254; UKD, *n* = 251) were collected. The median age was 42.0 years (IQR = 27.5–57.0 years; center range [across the eight clinics]: 39.0–44.0 years), with 53.9% (center range: 49.7–61.8%) of the patients being female (Table 1).

### 3.2. Diagnosis of MDD

Most patients had a (recurrent) severe depressive episode without psychotic symptoms (total: 65.8%; center range: 47.4%–86.0%), followed by a (recurrent) moderate depressive episode (total: 23.2%; center range: 6.6%–44.6%), a (recurrent) severe depressive episode with psychotic symptoms (total: 7.7%; center range: 1.6%–11.8%), other/unspecified depressive episode (total: 2.5; center range: 0.2%–7.9%), and a (recurrent) mild depressive episode (total: 0.9; center range: 0.2%–1.5%). Furthermore, 34.9% (center range: 25.1%–45.5%) of the patients had a first episode, while 65.1% (center range: 54.5%–74.9%) experienced a recurrent depressive episode (Table 1).

### 3.3. Comorbid Psychiatric Diagnoses

Altogether, 46.2% (center range: 24.2%–61.7%) of the patients had comorbid psychiatric diagnoses, with a mean of 1.6 (SD = 0.9; center mean range: 1.3–1.9). The most common comorbid diagnoses were substance use disorders (total: 18.9%; center range: 7.3%–33.3%), with 7.9% (center range: 1.0%–19.1%) of all patients having more than one substance use disorder. Among all patients, alcohol use disorder was the most prevalent (11.9%; center range: 5.1%–20.2%), while 10.6% (center range: 3.0%–21.4%) had other substance use disorders. Moreover, 8.4% (center range: 5.9%–10.5%) exhibited personality disorders, with the majority presenting with borderline personality disorder (total: 5.9%; center range: 3.7%–7.2%). Additionally, 7.6% (center range: 3.0%–14.1%) of the patients demonstrated a severe stress and adjustment disorder, whereby 6.1% (center range: 2.2%–10.9%) had a PTSD. Furthermore, 6.6% (center range: 0.5%–16.8%) of the patients exhibited phobic and other anxiety disorders (Table 2).

### 3.4. Suicidality

Upon admission, 72.0% (center range: 45.1%–85.2%) of patients reported experiencing passive or active suicidal thoughts. In detail, 25.3% (center range: 17.3%–41.2%) of patients exhibited passive suicidal thoughts, 17.2% (center range: 8.3%–30.3%) demonstrated active suicidal thoughts without a plan, 15.0% (center range: 1.1%–27.4%) displayed active suicidal thoughts with a plan and intention, and 14.5% (center range: 5.2%–19.6%) exhibited active suicidal thoughts with a plan. A total of 11.5% (center range: 4.6%–17.1%) of patients had attempted suicide within the 2-week period preceding admission. During lifetime, 83.9% (center range: 75.5%–94.3%) of patients had passive or active suicidal ideation, with 36.0% (center range: 23.3%–47.8%) having attempted suicide and a mean number of suicide attempts of 1.9 (center range: 1.0%–2.5%; Table 3).

### 3.5. Treatment Prior to Inpatient Admission

A lifetime history of prior inpatient treatment for MDD was documented in 57.8% (center range: 49.2%–65.7%) of cases. Among all patients, 20.8% (center range: 12.1%–26.1%) had undergone one inpatient treatment, while 29.0% (center range: 23.7%–40.7%) had two to five, 6.0% (center range: 2.5%–10.8%) had six to ten, and 2.0% (center range: 0.0%–3.7%) had more than 10 prior psychiatric inpatient treatments for MDD.

Furthermore, 62.3% (center range: 53.4%–74.7%) of patients had received outpatient psychotherapeutic or psychiatric treatment in the last 6 months, with a lifetime history in 76.8% (center range: 66.7%–87.3%). Finally, 4.0% (center range: 0.8%–9.4%) of patients had undergone ECT in their lifetime (Table 3).

### 3.6. Inpatient Admission Context

Referrals were self-initiated in 54.9% (center range: 29.5–70.5%) of cases, followed by outpatient care provider in 13.3% (center range: 3.4%–33.6%), inpatient care provider in 8.7% (center range: 4.1%–12.4%), family or friends in 8.4% (center range: 3.3%–11.9%), ambulance in 7.7% (center range: 0.8%–13.6%), police in 3.2% (center range: 0.0%–8.1%), and other referral context in 4.1% (center range: 1.2%–8.8%) of cases.

Altogether, 42.5% (center range: 11.8%–80.5%) of patients were admitted due to a psychiatric emergency, with suicidality being identified as the primary reason in 35% (center range: 23.0%–89.3%) of cases, followed by stupor/refusal or intoxication or acute agitation (0.9%–1.5%), or danger to others or delirium (0.1%–0.3%; Table 3).

### 3.7. TRD

A total of 18.2% (center range: 3.9%–39.9%) of patients experienced TRD in their lifetime (Table 1).

### 3.8. Pharmacological Treatment at Admission

Upon admission, 71.6% (center range: 64.8%–95.7%) of patients received psychopharmacological treatment, with a mean of 1.2 (SD = 1.1; center mean range: 0.9–1.3) medications per patient. The overall mean number of medications for all patients' prescribed pharmacological treatment was 1.8 (SD = 0.9; center mean range: 1.5–2.1). The number of prescribed psychiatric medications was as follows: 26.4% (center range: 5.6%–51.7%) of patients received one medication, 20.2% (center range: 0.2%–33.3%) two medications, 13.5% (center range: 7.2%–25.9%) three medications, and 9.1% (center range: 1.3%–33.5%) received more than three medications (Table 4).

### 3.9. Antidepressant Treatment at Admission

Upon admission, 59.8% (center range: 45.3%–80.8%) of patients were treated with antidepressants, with a mean of 0.7 (SD = 0.7; center mean range: 0.6–1.0) antidepressants per patient. The number of prescribed antidepressants was as follows: 44.8% (center range: 34.4%–64.5%) received one medication, 13.6% (center range: 8.5%–25.7%) two medications, 1.2% (center range: 0.4%–1.6%) three medications, and 0.1% (center range: 0.0%–0.4%) more than three medications. Among the various classes of antidepressants, SSRI were prescribed in 27.4% of patients (center range: 20.6%–44.4%), SNRI in 17.6% (center range: 11.4%–23.8%), noradrenergic and specific serotonergic antidepressant (NaSSa) in 13.7% (center range: 10.8%–21.8%), TCA in 5.8% (center range: 3.3%–6.8%), and monoamine-oxidase (MAO) inhibitors in 1.0% (center range: 0.0%–2.5%; Table 4).

### 3.10. Mood Stabilizer Treatment at Admission

Upon admission, 8.1% (center range: 4.6%–12.7%) of patients were treated with mood stabilizers, with a mean of 0.1 (SD = 0.3; center mean range: 0.0–0.4) mood stabilizers per patient. The number of prescribed mood stabilizers was as follows: 8.1% (center range: 4.6%–12.7%) of patients received one medication, 0.4% (center range: 0.0%–1.2%) two medications, 0.1% (center range: 0.0%–0.4%) three medications, and 0.1% (center range: 0.0%–0.4%) more than three medications. Among the various classes of mood stabilizers, anticonvulsants were prescribed in 4.0% (center range: 0.0%–10.8%), and lithium in 3.8% (center range: 2.3%–6.4%).

### 3.11. Antipsychotic Treatment at Admission

Upon admission, 25.1% (center range: 20.1%–32.4%) of patients received antipsychotics, with a mean of 0.3 (SD = 0.5; center mean range: 0.2–0.4) antipsychotics per patient. The number of prescribed antipsychotics was as follows: 21.5% (center range: 17.8%–27.1%) of patients received one medication, 3.4% (center range: 1.7%–5.1%) two medications, 0.3% (center range: 0.0%–0.9%) three medications, and 0.0% (center range: 0.0%) more than three medications. Among the various classes of antipsychotics, second-generation antipsychotics were prescribed in 19.0% (center range: 0.0%–31.4%), and first-generation antipsychotics in 5.3% (center range: 1.6%–13.3%; Table 5).

### 3.12. Anxiolytics/Hypnotics and Other Psychiatric Medication Treatment at Admission

Upon admission, 11.8% (center range: 3.0%–26.7%) of patients were on anxiolytics/hypnotics medication, with a mean of 0.1 (SD = 0.3; center mean range: 0.1–0.3) and 4.4% (center range: 1.9%–10.3%) of patients were administered other psychiatric medication (Table 5).

### 3.13. Duration of Inpatient Treatment

The median duration of inpatient treatment was 31.0 (IQR = 13.0, 57.0) days (mean provided for comparison with prior studies: 40.0 days (SD = 37.8; center mean range: 20.2–68.6; Table 1).

## 4. Discussion

This study of 3795 patients hospitalized with a single or recurrent depressive episode yielded the following main results: (i) the median age of the sample was 42 years and little more than half of the patients were female; (ii) the depressive episode was severe in three out of four cases (without psychotic symptoms: 67%, with psychosis: 8%); (iii) one-third had a first episode of MDD; (iv) one-fifth had TRD; (v) about half had psychiatric comorbidities, mostly consisting of substance use disorders, personality disorders, reaction to severe stress/adjustment disorders, and phobic/other anxiety disorders; (vi) a psychiatric emergency was a common reason for hospitalization, mainly due to suicidality; (vii) almost three-fourth had active or passive suicidal thoughts upon admission, 12% had attempted suicide within 2 weeks prior to admission, and 36% had a lifetime suicide attempt; (viii) 72% were prescribed psychiatric medications, but only <60% received antidepressants at time of admission; (ix) lifetime history of ECT was recorded in 4%; (x) and the median length of inpatient stay was about 40 days.

The median age of the sample was 42.0 years, aligning with the range of mean ages observed in other studies of hospitalized patients with MDD, which spanned from 34.4 to 52.0 years [17, 22–28, 30, 31]. While this study found a comparatively low proportion of female patients compared to other studies (53.9 vs. 53.1%–73.0% [17–29]), studies with larger numbers of patients tended to have less females (54.9%–63.2%) [17, 19–21, 23, 25, 29].

Compared with other studies, our findings revealed a relatively high prevalence of severe depression without psychotic symptoms (65.8% vs. 39.6%–72.1% [17, 20, 23, 32–34]), a relatively low prevalence of severe depression with psychotic symptoms (7.7% vs. 7.1%–25.9% [17, 20, 23, 32–34]), and a lower proportion of patients with moderate depressive episodes (23.2% vs. 28.1%–57.9% [17, 20, 23, 32–34]). Furthermore, first depressive episodes were present considerably less frequently in this study compared to other studies (34.9% vs. 51.0%–83.9% [17, 26, 27, 33–36]), whereas recurrent depressive episodes were present more frequently (65.1% vs. 16.1%–49.0% [17, 26, 27, 33–36]). The higher rate of severe and/or recurrent depressive disorders indicates referral paths and treatment settings geared more toward more severely ill patients in our study.

The prevalence of TRD was 18.2%, which was on the lower end of figures from prior studies (14.6%–29.3%) [24, 32]. However, the prevalence may be underestimated due to missing data regarding TRD at admission in 19.7% of patients.

Altogether, 46.2% of the sample had at least one psychiatric comorbidity (mean 1.6 ± 0.9), which is higher compared to 23.8%–42.5% [37–39] in two prior studies, while other studies reported only specific psychiatric diagnostic groups. Specific comorbid psychiatric disorders were similar to other studies, including substance use disorders (18.9% vs. 5.0%–22.9% [23–25, 28, 30, 34, 42]), other substance use disorders (10.6% vs. 1.8%–17.6% [23, 28, 30, 34, 42]), personality disorders (8.4% vs. 2.6%–23.1% [17, 23, 24, 27, 29, 37, 39, 41, 42]), and reaction to severe stress and adjustment disorders (7.6% vs. 6.5% [34]). However, the prevalence of PTSD was somewhat higher (6.1% vs. 3.7% [43]). In contrast, the prevalence of phobic and anxiety disorders was somewhat lower than in other studies (6.6% vs. 10.0%–41.8%) [23, 25, 27, 29, 34, 40–42, 55]. This discrepancy regarding phobic and anxiety disorders may be due to heterogeneity in group definitions. For example, studies included patients with a diagnosis of F40–F49 [29], F40–F48 [23], or F40–43 [42], whereas in one study with identical grouping (ICD-10: F40, F41), the proportion was nearly equal at 10.0% [55].

Psychiatric emergencies accounted for nearly half of the admissions (42.5%; 35.0% due to suicidality), which is comparable to the findings of a large Danish registry study (50.2%) [44]. While the prevalence of suicidal thoughts at admission was higher than in other studies (70.7% vs. 20.9%–63.0%) [22, 24, 25, 30, 36, 45, 46], frequencies were within the high end of the range of prior studies for lifetime suicidal ideation (83.6% vs. 67.0%–84.3% [47, 48]), suicide attempts before admission (11.6% vs. 3.7%–12.1% [24, 25]), and lifetime suicide attempts (35.7 vs. 15.4%–57.8% [23, 26, 45, 46, 48]). The relatively higher proportion of patients with suicidality matches the relatively higher severity of the MDD episode in our sample compared to others in the literature.

In the OASIS-D study, 72% of patients were prescribed psychotropic medications, which is slightly lower compared to other studies where the rates ranged from 76.7% to 83.2% [30, 49]. Similarly, the average number of psychotropic medications per patient was slightly lower in our study (1.8 vs. 2.5) [32]. Antidepressants were prescribed at admission in 59.8% of patients, which is consistent with other studies (40.2%–78.1%) [22, 26, 28, 30]. Nevertheless, the prescription rates for all types of antidepressants were at the lower end compared to comparable studies: SSRI: 27.4% vs. 25.0%–49.0% [26, 28, 49, 50]; SNRI: 17.6% vs. 17.4%–34.5% [26, 28, 49, 50]; TCA 5.8% vs. 2.1%–26.2% [26, 28, 50]. These data suggest that in the international comparison, German ambulatory care may be associated with lower use of antidepressants in adults diagnosed with MDD. Whether this situation is due to greater reliance on and delivery of psychotherapeutic interventions, undertreatment or delayed treatment by prescribers, or more frequent refusal of antidepressants by patients requires further study.

While mood stabilizers were prescribed prior to admission at similar rates as in other studies (8.6% vs. 4.9%–16.5% [26, 28, 30, 34]), antipsychotics (25.1% vs. 36.4–60.1% [26, 28, 30, 34]) were prescribed less frequently, and, especially, anxiolytics/hypnotics were prescribed much less frequent than in one study reporting the rate of anxiolytics/hypnotics (11.8% (center range: 6.9%−26.7%) vs. anxiolytics: 32.3% (1995)–32.4% (2001) [60]; hypnotics: 18.0% (1995)–27.9% (2001) [60]).

A lifetime history of ECT was documented in 4.0% of patients, a frequency that is somewhat below the range reported in other studies (5.5%–22.5%) [23, 51–54]. However, the frequency of ECT during inpatient treatment was included in these studies, which may have resulted in an overestimation of the frequency of patients with exposure to ECT. Conversely, now all patients in our study were asked about a lifetime history of ECT (21.2% with missing data). Moreover, only in Finland [23] and Sweden [54] did frequencies exceed 9%, whereas lower frequencies were reported in Canada (5.5%) [52], Denmark (7.4%) [53], and Australia/New Zealand (6%) [51].

Overall, the average inpatient stay of 40.0 ± 37.8 days (median: 31 days) in our study, is in the higher range compared to other studies (mean duration range from 8.6 to 70 days and two studies reported median durations of 26 and 35 days) [20, 22, 26, 27, 38, 55–57, 61]. However, the mean length of stay (LOS) in Germany appears to be longer than in other European countries, such as Switzerland, France, and Austria (18–37 days) [20, 26, 56]. Furthermore, countries like South Korea, the USA, Australia, and China, tend to treat patients hospitalized for MDD with a much shorter mean LOS, typically <20 days [27, 57, 61]. These differences reflect structural or procedural variations in healthcare access and delivery. Additionally, the range of mean LOS across the centers in our study is quite broad (20.2–68.6 days). However, six of the eight centers had mean LOS raging between 32 and 47 days. The outlier centers with especially short LOS may be explained by implementation of track system enabling efficient transfer from inpatient to outpatient settings [62], despite treating patients with one of the highest severities and treatment resistance proportions in this study for the center with the shortest LOS. In contrast, the longest LOS in one center may be explained by a focus on patients with especially complex MDD, more prior ECT treatment, and with referrals not only through the emergency department.

Differences between centers were evident, for example, regarding the proportion of patients with depressive episodes, first versus recurrent episodes, and with psychiatric comorbidities, especially substance use disorders. Variation was also observed in admission pathways, with some centers reporting more emergency admissions and suicidality at intake, as well as in treatment characteristics or LOS. Such heterogeneity likely reflects regionally differences in structural factors within the German health care system, including the presence and characteristics of acute psychiatric units, specialized wards, and differences in regional catchment areas and outpatient resources. These institutional aspects should be considered when interpreting the generalizability of the findings.

Taken together, the results of this study provide valuable insights into the characteristics and treatment of patients hospitalized with unipolar depressive episodes. The findings highlight the severe nature of depression in this cohort, as evidenced by the high prevalence of severe depressive episodes, significant psychiatric comorbidities, and substantial suicidal ideation and attempts. The data also indicate a relatively lower rate of psychotropic medication prescriptions and a duration of inpatient stays on the longer end of the range of prior studies. Additionally, the frequency of lifetime ECT was relatively low, despite the high number of patients with prior episodes and about one fifth of patients with TRD. These results indicate that the patients in the present study may have been treated with a more conservative psychosocial and/or psychopharmacological approach.

This study has several limitations. First, data for the baseline population of the OASIS-D study were extracted retrospectively from routine clinical practice, which may have resulted in an incomplete and/or inaccurate data set. Nevertheless, data were abstracted from the medical records using a standardized and detailed data capture form. Second, ICD-10 diagnoses were based on clinical decision, which can lead to heterogeneity in assigning MDD and comorbid psychiatric diagnoses. Third, with regard to the study design, data were transferred to Charité–Universitätsmedizin Berlin only in aggregated form, which precluded sub-analyses at the individual level across all eight participating clinics. Fourth, data for the population 1 of the OASIS-D study were only assessed at baseline, and outcome results are missing. However, prospective data were collected and will be reported as part of the longitudinal OASIS-D cohort of patients with research interview-verified diagnosis of moderate or severe MDD and documented suicidality for ≥48 h after admission who consented to participation in that part of the study [58]. Fifth, important information was not collected, including socioeconomic status, measures of psychopathology, medical comorbidities, involuntary admission, and QOL, outcomes that are included in the prospective data collection of OASIS-D. Sixth, regional differences in healthcare practices may limit the generalizability of the findings. Seventh, our analyses are restricted to hospitalized patients with MDD, and no direct comparison with the non-hospitalized patients with MDD are possible. Eighth, the data were based solely on clinicians' perspectives; ratings by patients and caregivers were not included that are important when describing the outcomes of patients living with MDD.

Nevertheless, despite these limitations, this study provided a comprehensive picture of a large sample of consecutively admitted adults with a unipolar depressive episode under real-world conditions. Results suggest that hospitalized adults with MDD are suffering from mostly severe and recurrent MDD that is characterized by suicidality, which often was the emergency reason for the hospitalization. Together with the finding that only less than 60% of the hospitalized sample received antidepressants at time of admission, these findings point to the need for early and comprehensive screening for and identification of MDD in the community, as well as for targeted treatment and a focus on suicidality. Additional analyses in the OASIS-D sample are underway to compare clinical and research diagnoses at time of admission and to follow patients who had continued suicidality of ≥48 h post-admission during in-patient and outpatient care to assess symptomatic and functional outcomes and their correlates.

In order to represent an even larger and more complete and ideally representative picture of people living with MDD and other psychiatric disorders who receive treatment in usual care settings, registries like in some Scandinavian countries should be initiated more broadly, including in Germany [63]. Such registries that overcome the limitations presented by multiple health care insurance partners, like present in Germany, should collect data with interoperable methods but also enrich the routine registries by including structed patient reported and clinician reported outcomes that are particularly important in mental health [63–65]. Integrating patient and caregiver perspectives can provide a more holistic view of the impact of unipolar depression and enhance the accuracy of the data [64, 66]. Furthermore, standardizing diagnostic assessment tools across regions can help mitigate heterogeneity and improve the generalizability of diagnoses and treatment approaches [64]. These improvements are hoped to pave the way towards measurement-based care in psychiatry and ultimately contribute to more robust and reliable research, guiding better clinical practices, and improving patient outcomes [67].

## 5. Conclusions

Current analyses indicate that the baseline sample of the OASIS-D study included a relatively ill patient population having predominantly severe and recurrent MDD and prominent suicidality with almost half of the sample having been admitted as a psychiatric emergency. Comorbid psychiatric disorders were the rule. Despite likely ongoing depression prior to hospitalization, less than 60% of the patients requiring an inpatient treatment for MDD received an antidepressant at time of admission, indicating under-recognition and/or undertreatment. Early identification and comprehensive care of patients with an MDD episode, especially those with suicidal ideation, both prior to and after hospital care is required to improve overall outcomes.

## Figures and Tables

**Table 1:** Demographic characteristics, primary diagnosis, and duration of inpatient treatment of adults hospitalized with a major depressive episode.

Characteristic	Overall (*n* = 3795)	Charité–St. Hedwig (*n* = 817)	Uni Köln (*n* = 665)	KGU (*n* = 607)	LMU (*n* = 526)	Charité–Mitte (*n* = 405)	ZI Mannheim (*n* = 270)	Charité–CBF (*n* = 254)	UKD (*n* = 251)
Demographic information									
Age, median (IQR)	42.0 (27.5, 57.0) (*n* = 3794)	42.0 (27.0, 52.0) (*n* = 817)	42.0 (27.0, 57.0) (*n* = 665)	42.0 (27.0, 52.0) (*n* = 607)	44.0 (28.0, 58.0) (*n* = 525)	42.0 (32.0, 57.0) (*n* = 405)	39.0 (28.0–53.0) (*n* = 270)	42.0 (32.0, 57.0) (*n* = 254)	42.0 (27.0, 57.0) (*n* = 251)
Sex, *n* (%)	*n* = 3793	*n* = 817	*n* = 665	*n* = 606	*n* = 525	*n* = 405	*n* = 270	*n* = 254	*n* = 251
Female	2043 (53.9)	406 (49.7)	349 (51.7)	341 (56.3)	292 (55.6)	222 (54.8)	140 (51.9)	157 (61.8)	136 (54.2)
Male	1741 (45.9)	410 (50.2)	316 (46.8)	265 (43.7)	231 (43.9)	183 (45.2)	129 (47.8)	95 (37.4)	112 (44.6)
Diverse	9 (0.2)	1 (0.1)	0 (0.0)	0 (0.0)	2 (0.4)	0 (0.0)	1 (0.4)	2 (0.8)	3 (1.2)
Primary diagnosis									
Diagnosis of MDD	*n* = 3795	*n* = 817	*n* = 665	*n* = 607	*n* = 526	*n* = 405	*n* = 270	*n* = 254	*n* = 251
Severe without psychotic symptoms	2496 (65.8)	499 (61.1)	445 (66.9)	522 (86.0)	438 (83.3)	192 (47.4)	150 (55.6)	133 (52.4)	117 (46.6)
Moderate	880 (23.2)	176 (21.5)	143 (21.5)	40 (6.6)	40 (7.6)	155 (38.3)	108 (40.0)	106 (41.7)	112 (44.6)
Severe with psychotic symptoms	293 (7.7)	96 (11.8)	62 (9.3)	42 (6.9)	46 (8.7)	20 (4.9)	9 (3.3)	4 (1.6)	14 (5.6)
Mild	33 (0.9)	11 (1.3)	6 (0.9)	1 (0.2)	1 (0.2)	6 (1.5)	2 (0.7)	3 (1.2)	3 (1.2)
Other/unspecified depressive episode	93 (2.5)	35 (4.3)	9 (1.4)	2 (0.3)	1 (0.2)	32 (7.9)	1 (0.4)	8 (3.1)	5 (2.0)
First vs. recurrent MDD	*n* = 3795	*n* = 817	*n* = 665	*n* = 607	*n* = 526	*n* = 405	*n* = 270	*n* = 254	*n* = 251
Recurrent depression	2472 (65.1)	445 (54.5)	395 (59.4)	446 (73.5)	390 (74.1)	231 (57.0)	190 (70.4)	187 (73.6)	188 (74.9)
First episode	1323 (34.9)	372 (45.5)	270 (40.6)	161 (26.5)	136 (25.9)	174 (43.0)	80 (29.6)	67 (26.4)	63 (25.1)
Treatment-resistant depression	*n* = 3048	*n* = 519	*n* = 586	*n* = 437	*n* = 429	*n* = 371	*n* = 206	*n* = 252	*n* = 248
Yes	554 (18.2)	20 (3.9)	78 (13.3)	60 (13.7)	116 (27.0)	148 (39.9)	74 (35.9)	45 (17.9)	13 (5.2)
No	2494 (81.8)	499 (96.1)	508 (86.7)	377 (86.3)	313 (73.0)	223 (60.1)	132 (64.1)	207 (82.1)	235 (94.8)
Duration of inpatient treatment									
Duration of inpatient treatment (days), median (IQR)	31.0 (13.0, 57.0) (*n* = 3396)	22.0 (9.0, 49.0) (*n* = 792)	36.0 (21.0, 61.0) (*n* = 443)	39.0 (15.0, 66.0) (*n* = 614)	38.0 (18.0, 63.5) (*n* = 452)	23.0 (8.0, 42.0) (*n* = 405)	16.5 (9.0, 25.0) (*n* = 270)	63.0 (38.0, 87.0) (*n* = 245)	26.0 (14.0, 47.0) (*n* = 175)
Duration of inpatient treatment (days), mean (SD)^a^	40.0 (37.8) (*n* = 3396)	33.3 (35.1) (*n* = 792)	44.4 (35.1) (*n* = 443)	44.4 (36.3) (*n* = 614)	47.4 (43.8) (*n* = 452)	32.2 (38.0) (*n* = 405)	20.2 (17.4) (*n* = 270)	68.6 (42.8) (*n* = 245)	33.2 (26.0) (*n* = 175)

*Note*: Charité–CBF, Charité Campus Benjamin Franklin, Department of Psychiatry and Psychotherapy; Charité–Mitte, Charité Campus Mitte, Department of Psychiatry and Psychotherapy; Charité–St. Hedwig, St. Hedwig Hospital, Department of Psychiatry and Psychotherapy; KGU, Department of Psychiatry, Psychosomatic Medicine and Psychotherapy at the University Hospital Frankfurt; LMU, Clinic for Psychiatry and Psychotherapy at the Ludwig-Maximilians-University Munich; UKD, Department of Psychiatry and Psychotherapy at the University Hospital Carl Gustav Carus in Dresden; Uni Köln, Department of Psychiatry and Psychotherapy at the University Hospital of Cologne; ZI Mannheim, Central Institute of Mental Health, Department of Psychiatry and Psychotherapy in Mannheim.

Abbreviations: IQR, interquartile range; SD, standard deviation.

^a^Means provided despite non-normally distributed data to allow for comparison with prior studies that mostly reposted means rather than medians.

**Table 2:** Psychiatric comorbid diagnoses of adults hospitalized with a major depressive episode.

Characteristic	Overall (*n* = 3795)	Charité–St. Hedwig (*n* = 817)	Uni Köln (*n* = 665)	KGU (*n* = 607)	LMU (*n* = 526)	Charité–Mitte (*n* = 405)	ZI Mannheim (*n* = 270)	Charité–CBF (*n* = 254)	UKD (*n* = 251)
Comorbid diagnosis^a^	*n* = 3718	*n* = 802	*n* = 664	*n* = 588	*n* = 492	*n* = 409	*n* = 256	*n* = 255	*n* = 252
Yes	1718 (46.2)	447 (55.7)	275 (41.4)	283 (48.1)	119 (24.2)	196 (47.9)	158 (61.7)	116 (45.5)	124 (49.2)
Number of diagnosis, mean (SD)	1.6 (0.9)	1.9 (1.1)	1.4 (0.8)	1.7 (0.9)	1.3 (0.5)	1.6 (1.0)	1.7 (0.9)	1.5 (0.9)	1.7 (1.0)
Substance use disorder, *n* (%)	702 (18.9)	267 (33.3)	105 (15.8)	114 (19.4)	36 (7.3)	69 (16.9)	30 (11.7)	26 (10.2)	55 (21.8)
>1 substance use disorder	292 (7.9)	153 (19.1)	34 (5.1)	39 (6.6)	5 (1.0)	26 (6.4)	9 (3.5)	6 (2.4)	20 (7.9)
Alcohol use disorder	442 (11.9)	162 (20.2)	68 (10.2)	68 (11.6)	26 (5.3)	43 (10.5)	20 (7.8)	13 (5.1)	42 (16.7)
Other use disorder	393 (10.6)	172 (21.4)	52 (7.8)	60 (10.2)	15 (3.0)	37 (9.0)	14 (5.5)	17 (6.7)	26 (10.3)
Cannabinoid use disorder	143 (3.8)	75 (9.4)	6 (0.9)	24 (4.1)	4 (0.8)	7 (1.7)	7 (2.7)	7 (2.7)	13 (5.2)
Sedatives/hypnotics use disorder	137 (3.7)	54 (6.7)	16 (2.4)	24 (4.1)	6 (1.2)	19 (4.6)	4 (1.6)	8 (3.1)	6 (2.4)
Opioid use disorder	86 (2.3)	47 (5.9)	8 (1.2)	16 (2.7)	2 (0.4)	4 (1.0)	4 (1.6)	1 (0.4)	4 (1.6)
Cocaine use disorder	60 (1.6)	32 (4.0)	7 (1.1)	14 (2.4)	1 (0.2)	3 (0.7)	1 (0.4)	1 (0.4)	1 (0.4)
Stimulant use disorder	41 (1.1)	26 (3.2)	1 (0.2)	6 (1.0)	1 (0.2)	1 (0.2)	2 (0.8)	1 (0.4)	3 (1.2)
Hallucinogen use disorder	4 (0.1)	4 (0.5)	0 (0.0)	0 (0.0)	0 (0.0)	0 (0.0)	0 (0.0)	0 (0.0)	0 (0.0)
Personality disorder	314 (8.4)	69 (8.6)	57 (8.6)	52 (8.8)	32 (6.5)	36 (8.8)	27 (10.5)	15 (5.9)	26 (10.3)
Emotionally unstable personality disorder, borderline type	220 (5.9)	52 (6.5)	48 (7.2)	39 (6.6)	18 (3.7)	23 (5.6)	15 (5.9)	11 (4.3)	14 (5.6)
Schizoid personality disorder	52 (1.4)	19 (2.4)	14 (2.1)	1 (0.2)	1 (0.2)	10 (2.4)	2 (0.8)	1 (0.4)	4 (1.6)
Mixed and other personality disorders	43 (1.2)	4 (0.5)	6 (0.9)	15 (2.6)	2 (0.4)	4 (1.0)	3 (1.2)	3 (1.2)	6 (2.4)
Anxious (avoidant) personality disorder	19 (0.5)	0 (0.0)	5 (0.8)	1 (0.2)	1 (0.2)	2 (0.5)	10 (3.9)	0 (0.0)	0 (0.0)
Emotionally unstable personality disorder: impulsive type	11 (0.3)	5 (0.6)	3 (0.5)	1 (0.2)	0 (0.0)	0 (0.0)	1 (0.4)	0 (0.0)	1 (0.4)
Obsessive-compulsive (anankastic) personality disorder	6 (0.2)	0 (0.0)	0 (0.0)	0 (0.0)	1 (0.2)	0 (0.0)	2 (0.8)	1 (0.4)	2 (0.8)
Histrionic personality disorder	3 (0.1)	1 (0.1)	1 (0.2)	0 (0.0)	0 (0.0)	1 (0.2)	0 (0.0)	0 (0.0)	0 (0.0)
Dissocial personality disorder	2 (0.1)	0 (0.0)	1 (0.2)	1 (0.2)	0 (0.0)	0 (0.0)	0 (0.0)	0 (0.0)	0 (0.0)
Paranoid personality disorder	1 (0)	0 (0.0)	0 (0.0)	0 (0.0)	1 (0.2)	0 (0.0)	0 (0.0)	0 (0.0)	0 (0.0)
Other specific personality disorder/Personality disorder not otherwise specified	5 (0.1)	0 (0.0)	3 (0.5)	0 (0.0)	0 (0.0)	0 (0.0)	0 (0.0)	1 (0.4)	1 (0.4)
Reaction to severe stress and adjustment disorder	282 (7.6)	72 (9.0)	29 (4.4)	66 (11.2)	15 (3.0)	25 (6.1)	36 (14.1)	18 (7.1)	21 (8.3)
Posttraumatic-stress disorder	227 (6.1)	61 (7.6)	23 (3.5)	53 (9.0)	11 (2.2)	14 (3.4)	28 (10.9)	18 (7.1)	19 (7.5)
Acute stress disorder	9 (0.2)	3 (0.4)	1 (0.2)	3 (0.5)	0 (0.0)	2 (0.5)	0 (0.0)	0 (0.0)	0 (0.0)
Adjustment disorder	30 (0.8)	6 (0.7)	5 (0.8)	0 (0.0)	2 (0.4)	8 (2.0)	8 (3.1)	0 (0.0)	1 (0.4)
Other reaction to severe stress	15 (0.4)	2 (0.2)	0 (0.0)	10 (1.7)	1 (0.2)	1 (0.2)	0 (0.0)	0 (0.0)	1 (0.4)
Phobic disorders and other anxiety disorders	245 (6.6)	4 (0.5)	51 (7.7)	43 (7.3)	15 (3.0)	41 (10.0)	43 (16.8)	22 (8.6)	26 (10.3)
Panic disorder (incl. agoraphobic with panic disorder)	127 (3.4)	40 (5.0)	19 (2.9)	18 (3.1)	8 (1.6)	10 (2.4)	20 (7.8)	6 (2.4)	6 (2.4)
Generalized anxiety disorder	101 (2.7)	12 (1.5)	26 (3.9)	14 (2.4)	4 (0.8)	16 (3.9)	12 (4.7)	6 (2.4)	11 (4.4)
Agoraphobia/social phobia/specific phobia	69 (1.9)	12 (1.5)	7 (1.1)	11 (1.9)	2 (0.4)	13 (3.2)	11 (4.3)	7 (2.7)	6 (2.4)
Anxiety, not otherwise specified	11 (0.3)	3 (0.4)	1 (0.2)	2 (0.3)	0 (0.0)	0 (0.0)	1 (0.4)	2 (0.8)	2 (0.8)
Mixed anxiety-depressive disorder	7 (0.2)	2 (0.2)	1 (0.2)	0 (0.0)	0 (0.0)	1 (0.2)	2 (0.8)	0 (0.0)	1 (0.4)
Attention-deficit and/or hyperactivity disorder	119 (3.2)	25 (3.1)	13 (2.0)	30 (5.1)	6 (1.2)	16 (3.9)	16 (6.3)	9 (3.5)	4 (1.6)
Dysthymia	113 (3)	18 (2.2)	7 (1.1)	6 (1.0)	2 (0.4)	7 (1.7)	40 (15.6)	31 (12.2)	2 (0.8)
Somatoform disorder	105 (2.8)	15 (1.9)	12 (1.8)	19 (3.2)	4 (0.8)	18 (4.4)	16 (6.3)	13 (5.1)	8 (3.2)
Eating disorder	90 (2.4)	8 (1.0)	8 (1.2)	27 (4.6)	7 (1.4)	14 (3.4)	10 (3.9)	8 (3.1)	8 (3.2)
Obsessive-compulsive disorder	89 (2.4)	12 (1.5)	20 (3.0)	14 (2.4)	12 (2.4)	10 (2.4)	9 (3.5)	6 (2.4)	6 (2.4)
Other comorbid diagnoses^a^	155 (4.2)	27 (3.4)	18 (2.7)	35 (6.0)	9 (1.8)	22 (5.4)	13 (5.1)	11 (4.3)	20 (7.9)
No	2000 (53.8)	355 (44.3)	389 (58.6)	305 (51.9)	373 (75.8)	213 (52.1)	98 (38.3)	139 (54.5)	128 (50.8)

*Note*: Charité–CBF, Charité Campus Benjamin Franklin, Department of Psychiatry and Psychotherapy; Charité–Mitte, Charité Campus Mitte, Department of Psychiatry and Psychotherapy; Charité–St. Hedwig, St. Hedwig Hospital, Department of Psychiatry and Psychotherapy; KGU, Department of Psychiatry, Psychosomatic Medicine and Psychotherapy at the University Hospital Frankfurt; LMU, Clinic for Psychiatry and Psychotherapy at the Ludwig-Maximilian-University Munich; UKD, Department of Psychiatry and Psychotherapy at the University Hospital Carl Gustav Carus in Dresden; Uni Köln, Department of Psychiatry and Psychotherapy at the University Hospital of Cologne; ZI Mannheim, Central Institute of Mental Health, Department of Psychiatry and Psychotherapy in Mannheim.

Abbreviations: IQR, interquartile range; SD, standard deviation.

^a^For details, see Table S2.

**Table 3:** Suicidality, previous treatment, and inpatient admission context of adults hospitalized with a major depressive episode.

Characteristic	Overall (*n* = 3795)	Charité–St. Hedwig (*n* = 817)	Uni Köln (*n* = 665)	KGU (*n* = 607)	LMU (*n* = 526)	Charité–Mitte (*n* = 405)	ZI Mannheim (*n* = 270)	Charité–CBF (*n* = 254)	UKD (*n* = 251)
Suicidality									
Suicidality, current	*n* = 3645	*n* = 776	*n* = 662	*n* = 550	*n* = 508	*n* = 386	*n* = 267	*n* = 248	*n* = 248
No suicidal thoughts	1018 (27.9)	133 (17.1)	238 (35.3)	97 (17.6)	279 (54.9)	57 (14.8)	59 (22.1)	95 (38.3)	60 (24.2)
Passive	924 (25.3)	177 (22.8)	189 (28.0)	146 (26.5)	126 (24.8)	75 (19.4)	110 (41.2)	43 (17.3)	58 (23.4)
Active without plan	626 (17.2)	162 (20.9)	88 (13.0)	97 (17.6)	42 (8.3)	87 (22.5)	81 (30.3)	23 (9.3)	46 (18.5)
Active with plan	529 (14.5)	150 (19.3)	100 (14.8)	108 (19.6)	29 (5.7)	66 (17.1)	14 (5.2)	19 (7.7)	43 (17.3)
Active with plan and intention	548 (15.0)	154 (19.8)	47 (7.0)	102 (18.5)	32 (6.3)	101 (26.2)	3 (1.1)	68 (27.4)	41 (16.5)
Suicide attempt, current (<2 weeks before admission)	*n* = 3671	*n* = 776	*n* = 661	*n* = 568	*n* = 519	*n* = 386	*n* = 263	*n* = 251	*n* = 250
Yes	422 (11.5)	101 (13.0)	72 (10.7)	97 (17.1)	24 (4.6)	59 (15.3)	25 (9.5)	17 (6.9)	27 (10.9)
No	3249 (88.5)	675 (87.0)	589 (87.3)	471 (82.9)	495 (95.4)	327 (84.7)	238 (90.5)	231 (93.1)	223 (89.9)
Suicidality, lifetime	*n* = 3013	*n* = 523	*n* = 619	*n* = 394	*n* = 498	*n* = 368	*n* = 193	*n* = 201	*n* = 217
No suicidal thoughts	487 (16.2)	68 (13.0)	149 (24.1)	30 (7.6)	122 (24.5)	21 (5.7)	33 (17.1)	37 (18.4)	27 (12.4)
Passive or active suicidality without suicide attempt	1442 (47.9)	205 (39.2)	280 (45.2)	185 (47)	260 (52.2)	220 (59.8)	79 (40.9)	96 (47.8)	117 (53.9)
Suicide attempt	1084 (36.0)	250 (47.8)	190 (30.7)	179 (45.4)	116 (23.3)	127 (34.5)	81 (42.0)	68 (33.8)	73 (33.6)
Number of suicide attempts, mean (SD)^a^	1.9 (1.8) (*n* = 1084)	1.9 (1.5) (*n* = 250)	1.9 (2.5) (*n* = 190)	2.0 (1.9) (*n* = 179)	1.6 (1.1) (*n* = 116)	1.8 (1.3) (*n* = 127)	1.5 (1.2) (*n* = 81)	1.7 (1.0) (*n* = 68)	2.3 (2.3) (*n* = 73)
Previous treatment									
Previous inpatient treatment for depressive episode	*n* = 3503	*n* = 709	*n* = 647	*n* = 531	*n* = 508	*n* = 371	*n* = 243	*n* = 248	*n* = 246
Yes (frequency)	2023 (57.8)	395 (55.7)	362 (54.6)	300 (56.5)	334 (65.7)	231 (62.3)	121 (49.8)	159 (64.1)	121 (49.2)
* *1	729 (20.8)	185 (26.1)	144 (21.7)	123 (23.2)	80 (15.7)	45 (12.1)	54 (22.2)	52 (20.9)	46 (18.7)
2–5	1015 (29.0)	180 (25.4)	165 (24.9)	126 (23.7)	180 (35.4)	151 (40.7)	59 (24.3)	88 (35.3)	66 (26.8)
6–10	209 (6.0)	18 (2.5)	37 (5.6)	38 (7.2)	55 (10.8)	26 (7.0)	8 (3.3)	19 (7.6)	8 (3.3)
>10	70 (2.0)	12 (1.7)	16 (2.4)	13 (2.4)	19 (3.7)	9 (2.4)	0 (0.0)	0 (0.0)	1 (0.4)
No	1480 (42.2)	314 (44.3)	285 (43.0)	231 (43.5)	174 (34.3)	140 (37.7)	122 (50.2)	89 (35.9)	125 (50.8)
Outpatient psychotherapeutic/psychiatric treatment during last 6 months before admission	*n* = 3397	*n* = 728	*n* = 625	*n* = 507	*n* = 461	*n* = 352	*n* = 234	*n* = 245	*n* = 245
Yes	2118 (62.3)	453 (62.2)	365 (58.4)	280 (55.2)	336 (72.9)	188 (53.4)	148 (63.2)	183 (74.7)	165 (67.3)
No	1279 (37.7)	275 (37.8)	260 (41.6)	227 (44.8)	125 (27.1)	164 (46.6)	86 (36.8)	62 (25.3)	80 (32.7)
Outpatient psychotherapeutic/psychiatric treatment, lifetime	*n* = 3421	*n* = 729	*n* = 629	*n* = 508	*n* = 471	*n* = 363	*n* = 240	*n* = 241	*n* = 240
Yes	2626 (76.8)	564 (77.4)	467 (74.2)	339 (66.7)	411 (87.3)	281 (77.4)	175 (72.9)	208 (86.3)	181 (75.4)
No	795 (23.2)	165 (22.6)	162 (25.8)	169 (33.3)	60 (12.7)	82 (22.6)	65 (27.1)	33 (13.7)	59 (24.6)
Electroconvulsive therapy, lifetime	*n* = 2989	*n* = 541	*n* = 559	*n* = 518	*n* = 460	*n* = 301	*n* = 126	*n* = 233	*n* = 251
Yes	120 (4.0)	9 (1.7)	26 (4.7)	25 (4.8)	22 (4.8)	11 (3.7)	3 (2.4)	22 (9.4)	2 (0.8)
No	2869 (96.0)	532 (98.3)	533 (95.3)	493 (95.2)	438 (95.2)	290 (96.3)	123 (97.6)	211 (90.6)	249 (99.2)
Inpatient admission context									
Referral context, *n* (%)	*n* = 3619	*n* = 802	*n* = 658	*n* = 543	*n* = 487	*n* = 397	*n* = 241	*n* = 250	*n* = 249
Self-referred	1986 (54.9)	345 (43.0)	462 (68.4)	298 (54.9)	266 (54.6)	244 (61.5)	170 (70.5)	127 (50.8)	74 (29.7)
Outpatient care provider	481 (13.3)	114 (14.2)	23 (3.4)	20 (3.7)	111 (22.8)	24 (6.0)	35 (14.5)	84 (33.6)	70 (28.1)
Inpatient care provider	315 (8.7)	100 (12.5)	53 (7.9)	39 (7.2)	37 (7.6)	31 (7.8)	10 (4.1)	14 (5.6)	31 (12.4)
Family/friends	304 (8.4)	69 (8.6)	57 (8.4)	49 (9.0)	58 (11.9)	32 (8.1)	8 (3.3)	12 (4.8)	19 (7.6)
Ambulance	277 (7.7)	65 (8.1)	50 (7.4)	74 (13.6)	4 (0.8)	36 (9.1)	6 (2.5)	10 (4.0)	32 (12.9)
Police	114 (3.2)	44 (5.5)	2 (0.3)	44 (8.1)	2 (0.4)	14 (3.5)	7 (2.9)	0 (0.0)	1 (0.4)
Other referral context	150 (4.1)	65 (8.1)	11 (1.6)	19 (3.5)	9 (1.8)	16 (4.0)	5 (2.1)	3 (1.2)	22 (8.8)
Psychiatric emergency at time of admission	*n* = 3619	*n* = 805	*n* = 659	*n* = 487	*n* = 513	*n* = 388	*n* = 262	*n* = 254	*n* = 251
Yes	1537 (42.5)	234 (29.1)	306 (45.3)	392 (80.5)	181 (35.3)	185 (47.7)	67 (25.6)	30 (11.8)	142 (56.6)
Suicidality	1268 (35.0)	183 (78.2)	220 (32.6)	350 (89.3)	150 (29.2)	161 (87.0)	60 (23.0)	28 (93.3)	116 (81.7)
Other self-harm (e.g., stupor and refusal of eat)	53 (1.5)	11 (4.7)	22 (3.3)	6 (1.5)	3 (0.6)	8 (4.3)	3 (1.1)	0 (0.0)	0 (0.0)
Intoxication	36 (1.0)	9 (3.8)	8 (1.2)	4 (1.0)	5 (1.0)	6 (3.2)	4 (1.5)	0 (0.0)	0 (0.0)
Acute agitation	31 (0.9)	11 (4.7)	4 (0.6)	10 (2.6)	3 (0.6)	2 (1.1)	0 (0.0)	1 (3.3)	0 (0.0)
Danger to others	12 (0.3)	7 (3)	0 (0.0)	3 (0.8)	0 (0.0)	1 (0.5)	0 (0.0)	1 (3.3)	0 (0.0)
Delirium	2 (0.1)	0 (0)	1 (0.1)	0 (0.0)	0 (0.0)	0 (0)	0 (0.0)	0 (0.0)	1 (0.7)
Other	120 (3.3)	12 (5.1)	51 (7.7)	11 (2.8)	14 (2.7)	7 (3.8)	0 (0.0)	0 (0.0)	25 (17.6)
No information	15 (0.4)	1 (0.4)	0 (0.0)	8 (2.0)	6 (1.2)	0 (0)	0 (0.0)	0 (0.0)	0 (0.0)
No	2082 (57.5)	571 (70.9)	353 (52.3)	95 (19.5)	332 (64.7)	203 (52.3)	195 (74.4)	224 (88.2)	109 (43.4)

*Note*: Charité–CBF, Charité Campus Benjamin Franklin, Department of Psychiatry and Psychotherapy; Charité–Mitte, Charité Campus Mitte, Department of Psychiatry and Psychotherapy; Charité–St. Hedwig, St. Hedwig Hospital, Department of Psychiatry and Psychotherapy; KGU, Department of Psychiatry, Psychosomatic Medicine and Psychotherapy at the University Hospital Frankfurt; LMU, Clinic for Psychiatry and Psychotherapy at the Ludwig-Maximilian-University Munich; UKD, Department of Psychiatry and Psychotherapy at the University Hospital Carl Gustav Carus in Dresden; Uni Köln, Department of Psychiatry and Psychotherapy at the University Hospital of Cologne; ZI Mannheim, Central Institute of Mental Health, Department of Psychiatry and Psychotherapy in Mannheim.

Abbreviation: SD, standard deviation.

^a^Based on the number of patients with at least one suicide attempt.

**Table 4:** Overall pharmacologic and antidepressant treatment at admission of adults hospitalized with a major depressive episode.

Characteristic	Overall (*n* = 3795)	Charité–St. Hedwig (*n* = 817)	Uni Köln (*n* = 665)	KGU (*n* = 607)	LMU (*n* = 526)	Charité–Mitte (*n* = 405)	ZI Mannheim (*n* = 270)	Charité–CBF (*n* = 254)	UKD (*n* = 251)
Psychiatric medication at admission (number of patients)	*n* = 3477	*n* = 739	*n* = 664	*n* = 567	*n* = 513	*n* = 234	*n* = 269	*n* = 251	*n* = 240
Yes, *n* (%)	2490 (71.6)	479 (64.8)	443 (66.7)	370 (65.3)	392 (76.4)	224 (95.7)	204 (75.8)	182 (72.5)	196 (81.7)
Mean of psychotropic medication of all patients^a^, mean (SD)	1.2 (1.1)	1.0 (1.0)	1.3 (1.2)	1.1 (1.2)	1.6 (1.1)	0.9 (1.0)	1.0 (0.9)	1.4 (1.2)^a^	1.6 (1.3)
Mean in patients on psychotropic medications^a^	1.8 (0.9)	1.7 (0.8)	1.9 (1.0)	1.8 (1.0)	2.0 (0.9)	1.6 (0.8)	1.5 (0.6)	1.9 (1.0)	2.1 (1.1)
1 Psychotropic medication^a^, *n* (%)	919 (26.4)	251 (34.0)	133 (20.0)	32 (5.6)	157 (30.6)	121 (51.7)	79 (29.4)	77 (30.7)^a^	69 (28.8)
2 Psychotropic medications^a^	704 (20.2)	158 (21.4)	142 (21.4)	1 (0.2)	132 (25.7)	78 (33.3)	64 (23.8)	59 (23.5)^a^	70 (29.2)
3 Psychotropic medications^a^	471 (13.5)	53 (7.2)	62 (9.3)	147 (25.9)	71 (13.8)	22 (9.4)	49 (18.2)	34 (13.5)^a^	33 (13.8)
>3 Psychotropic medications^a^	317 (9.1)	17 (2.3)	27 (4.1)	190 (33.5)	32 (6.2)	3 (1.3)	12 (4.5)	12 (4.8)^a^	24 (10.0)
Antidepressants	2080 (59.8)	413 (55.9)	301 (45.3)	328 (57.8)	339 (66.1)	189 (80.8)	187 (69.5)	151 (60.2)	172 (71.7)
Mean (SD)^a^	0.7 (0.7)	0.6 (0.7)	0.7 (0.7)	0.7 (0.7)	0.8 (0.7)	0.6 (0.7)	1.0 (0.9)	0.8 (0.7)	0.9 (0.7)
1 Antidepressant^a^, *n* (%)	1558 (44.8)	331 (44.8)	232 (34.4)	257 (45.3)	249 (48.5)	151 (64.5)	107 (39.8)	108 (43.0)^a^	123 (51.3)
2 Antidepressants^a^	473 (13.6)	78 (10.6)	57 (8.5)	61 (10.8)	82 (16.0)	36 (15.4)	69 (25.7)	42 (16.7)^a^	48 (20.0)
3 Antidepressants^a^	41 (1.2)	4 (0.5)	7 (1.0)	8 (1.4)	8 (1.6)	2 (0.9)	10 (3.7)	1 (0.4)^a^	1 (0.4)
>3 Antidepressants^a^	2 (0.1)	0 (0.0)	0 (0.0)	1 (0.2)	0 (0.0)	0 (0.0)	1 (0.4)	0 (0.0)^a^	0 (0.0)
SSRI^b^	953 (27.4)	207 (28.0)	137 (20.6)	153 (27.0)	143 (27.9)	104 (44.4)	74 (27.5)	57 (22.7)	78 (32.5)
Citalopram	131 (3.8)	30 (4.1)	22 (3.3)	25 (4.4)	20 (3.9)	11 (4.7)	7 (2.6)	8 (3.3)	8 (3.3)
Escitalopram	386 (11.1)	92 (12.4)	63 (9.5)	55 (9.7)	40 (7.8)	56 (23.9)	24 (8.9)	18 (7.5)	38 (15.8)
Fluoxetine	84 (2.4)	21 (2.8)	11 (1.7)	12 (2.1)	11 (2.1)	16 (6.8)	6 (2.2)	5 (2.1)	2 (0.8)
Paroxetine	43 (1.2)	7 (0.9)	9 (1.4)	7 (1.2)	7 (1.4)	3 (1.3)	3 (1.1)	5 (2.1)	2 (0.8)
Sertraline	330 (9.5)	57 (7.7)	53 (8.0)	58 (10.2)	59 (11.5)	19 (8.1)	34 (12.6)	21 (8.8)	29 (12.1)
Other	13 (0.4)	0 (0.0)	11 (1.7)	0 (0.0)	1 (0.2)	0 (0.0)	1 (0.4)	0 (0.0)	0 (0.0)
Fluvoxamine	1 (0.1)	0 (0.0)	–	0 (0.0)	–	0 (0.0)	1 (0.4)	0 (0.0)	0 (0.0)
No information	12 (0.3)	0 (0.0)	11 (1.7)	0 (0.0)	1 (0.2)	0 (0.0)	0 (0.0)	0 (0.0)	0 (0.0)
SNRIs^b^	611 (17.6)	84 (11.4)	95 (14.3)	119 (21.0)	103 (20.1)	42 (17.9)	64 (23.8)	48 (19.1)	56 (23.3)
Duloxetine	168 (4.8)	18 (2.4)	40 (6.0)	34 (6.0)	26 (5.1)	8 (3.4)	14 (5.2)	11 (4.6)	17 (7.1)
Venlafaxine (incl. Ret.)	406 (11.7)	63 (8.5)	68 (10.2)	81 (14.3)	51 (9.9)	34 (14.5)	41 (15.2)	32 (13.3)	36 (15.0)
Milnacipran	56 (1.6)	2 (0.3)	12 (1.8)	5 (0.9)	25 (4.9)	0 (0.0)	5 (1.9)	4 (1.7)	3 (1.3)
Other	16 (0.5)	1 (0.1)	12 (1.8)	0 (0.0)	1 (0.2)	0 (0.0)	0 (0.0)	1 (0.4)	1 (0.4)
Denvenlafaxine	7 (0.2)	1 (0.1)	–	0 (0.0)	–	0 (0.0)	4 (1.5)	1 (0.4)	1 (0.4)
No information	14 (0.4)	0 (0.0)	12 (1.8)	0 (0.0)	1 (0.2)	0 (0.0)	1 (0.4)	0 (0.0)	0 (0.0)
NaSSa^b^	476 (13.7)	81 (11.0)	76 (11.4)	63 (11.1)	90 (17.5)	51 (21.8)	38 (14.4)	26 (10.8)	51 (21.3)
Mirtazapine	501 (14.4)	84 (11.4)	96 (14.5)	63 (11.1)	89 (17.3)	51 (21.8)	38 (14.4)	26 (10.8)	54 (22.5)
Mianserin	3 (0.1)	0 (0.0)	1 (0.2)	0 (0.0)	1 (0.2)	1 (0.4)	0 (0.0)	0 (0.0)	0 (0.0)
Other	1 (0.1)	0 (0.0)	0 (0.0)	0 (0.0)	1 (0.2)	0 (0.0)	0 (0.0)	0 (0.0)	0 (0.0)
No information	1 (0.1)	0 (0.0)	0 (0.0)	0 (0.0)	1 (0.2)	0 (0.0)	0 (0.0)	0 (0.0)	0 (0.0)
Tricyclic antidepressants^b^	200 (5.8)	46 (6.2)	32 (4.8)	37 (6.5)	35 (6.8)	11 (4.7)	14 (5.2)	17 (6.8)	8 (3.3)
Amitriptyline	78 (2.2)	14 (1.9)	13 (2.0)	23 (4.1)	14 (2.7)	3 (1.3)	7 (2.6)	2 (0.8)	2 (0.8)
Clomipramine	9 (0.3)	1 (0.1)	0 (0.0)	2 (0.4)	3 (0.6)	0 (0.0)	1 (0.4)	2 (0.8)	0 (0.0)
Timipramine	40 (1.2)	6 (0.8)	2 (0.3)	5 (0.9)	8 (1.6)	4 (1.7)	4 (1.5)	10 (4.2)	1 (0.4)
Nortriptyline	5 (0.1)	0 (0.0)	1 (0.2)	0 (0.0)	2 (0.4)	0 (0.0)	2 (0.7)	0 (0.0)	0 (0.0)
Other	80 (2.3)	24 (3.2)	24 (3.6)	7 (1.2)	11 (2.1)	4 (1.7)	2 (0.7)	8 (3.3)	0 (0.0)
Doxepine	27 (0.8)	12 (1.6)	–	3 (0.5)	4 (0.8)	4 (1.7)	1 (0.4)	1 (0.4)	2 (0.8)
Opipramole	24 (0.7)	9 (1.2)	–	2 (0.4)	5 (1.0)	0 (0.0)	1 (0.4)	4 (1.7)	3 (1.3)
Imipramine	2 (0.1)	1 (0.1)	–	0 (0.0)	1 (0.2)	0 (0.0)	0 (0.0)	0 (0.0)	0 (0.0)
Tianeptin	7 (0.2)	2 (0.3)	–	2 (0.4)	0 (0.0)	0 (0.0)	0 (0.0)	3 (1.3)	0 (0.0)
Amioxid	0 (0.0)	0 (0.0)	–	0 (0.0)	0 (0.0)	0 (0.0)	0 (0.0)	0 (0.0)	0 (0.0)
No information	25 (0.7)	0 (0.0)	24 (3.6)	0 (0.0)	1 (0.2)	0 (0.0)	0 (0.0)	0 (0.0)	0 (0.0)
MAO inhibitors^b^	34 (1.0)	1 (0.1)	6 (0.9)	2 (0.4)	12 (2.3)	4 (1.7)	0 (0.0)	3 (1.2)	6 (2.5)
Phenelzin	1 (0.1)	0 (0.0)	0 (0.0)	0 (0.0)	1 (0.2)	0 (0.0)	0 (0.0)	0 (0.0)	0 (0.0)
Trancylpromine (Jatrosome)	25 (0.7)	0 (0.0)	4 (0.6)	2 (0.4)	10 (1.9)	3 (1.3)	0 (0.0)	3 (1.3)	3 (1.3)
Other	11 (0.3)	1 (0.1)	4 (0.6)	0 (0.0)	1 (0.2)	1 (0.4)	0 (0.0)	1 (0.4)	3 (1.3)
Moclobemide	10 (0.3)	1 (0.1)	4 (0.6)	0 (0.0)	0 (0.0)	1 (0.4)	0 (0.0)	1 (0.4)	3 (1.3)
No information	1 (0.1)	0 (0.0)	0 (0.0)	0 (0.0)	1 (0.2)	0 (0.0)	0 (0.0)	0 (0.0)	0 (0.0)
Bupropion	162 (4.7)	35 (4.7)	21 (3.2)	12 (2.1)	33 (6.4)	12 (5.1)	29 (10.8)	16 (6.7)	4 (1.7)
Agomelatine	54 (1.6)	11 (1.5)	5 (0.8)	7 (1.2)	8 (1.6)	0 (0.0)	7 (2.6)	7 (2.9)	9 (3.8)
Vortioxetine	5 (0.1)	0 (0.0)	0 (0.0)	0 (0.0)	3 (0.6)	1 (0.4)	0 (0.0)	1 (0.4)	0 (0.0)
Vilazodon	0 (0.1)	0 (0.0)	0 (0.0)	0 (0.0)	0 (0.0)	0 (0.0)	0 (0.0)	0 (0.0)	0 (0.0)
Trazodone	118 (3.4)	26 (3.5)	0 (0.0)	7 (1.2)	9 (1.8)	0 (0.0)	50 (18.6)	16 (6.7)	10 (4.2)
Moclobomite	1 (0.1)	0 (0.0)	0 (0.0)	1 (0.2)	0 (0.0)	0 (0.0)	0 (0.0)	0 (0.0)	0 (0.0)
Escetamine	1 (0.1)	0 (0.0)	0 (0.0)	0 (0.0)	0 (0.0)	0 (0.0)	0 (0.0)	0 (0.0)	1 (0.4)
Other antidepressants^b^	14 (0.4)	5 (0.7)	2 (0.3)	0 (0.0)	0 (0.0)	6 (2.6)	0 (0.0)	0 (0.0)	1 (0.4)
St. John´s Wort	12 (0.3)	5 (0.7)	0 (0.0)	0 (0.0)	0 (0.0)	6 (2.6)	0 (0.0)	0 (0.0)	1 (0.4)
No information	2 (0.1)	0 (0.0)	2 (0.3)	0 (0.0)	0 (0.0)	0 (0.0)	0 (0.0)	0 (0.0)	0 (0.0)

*Note*: Charité–CBF, Charité Campus Benjamin Franklin, Department of Psychiatry and Psychotherapy; Charité–Mitte, Charité Campus Mitte, Department of Psychiatry and Psychotherapy; Charité–St. Hedwig, St. Hedwig Hospital, Department of Psychiatry and Psychotherapy; KGU, Department of Psychiatry, Psychosomatic Medicine and Psychotherapy at the University Hospital Frankfurt; LMU, Clinic for Psychiatry and Psychotherapy at the Ludwig-Maximilian-University Munich; UKD, Department of Psychiatry and Psychotherapy at the University Hospital Carl Gustav Carus in Dresden; Uni Köln, Department of Psychiatry and Psychotherapy at the University Hospital of Cologne; ZI Mannheim, Central Institute of Mental Health, Department of Psychiatry and Psychotherapy in Mannheim.

Abbreviations: IQR, interquartile range; MAO, monoamine-oxidase inhibitors; NaSSa, noradrenergic and specific serotonergic antidepressant; SD, standard deviation; SNRI, serotonin–norepinephrine reuptake inhibitors; SSRI, selective serotonin reuptake inhibitors.

^a^Data from the Uni Köln is only available starting from patient 114 in the clinic.

^b^The sum of patients on individual medications can be larger than the number of patients per medication class due to co-treatment with more than one agent.

**Table 5:** Non-antidepressant pharmacologic treatment at admission of adults hospitalized with a major depressive episode.

Characteristic	Overall (*n* = 3795)	Charité–St. Hedwig (*n* = 817)	Uni Köln (*n* = 665)	KGU (*n* = 607)	LMU (*n* = 526)	Charité–Mitte (*n* = 405)	ZI Mannheim (*n* = 270)	Charité–CBF (*n* = 254)	UKD (*n* = 251)
Mood stabilizers, *n* (%)	299 (8.6)	34 (4.6)	62 (9.3)	36 (6.3)	49 (9.6)	27 (11.5)	23 (8.6)	35 (13.9)	33 (13.8)
Mean (SD)^a^	0.1 (0.3)	0.1 (0.2)	0.1 (0.3)	0.1 (0.2)	0.1 (0.3)	0.1 (0.3)	0.4 (0.1)	0.2 (0.4)	0.1 (0.4)
1 Mood stabilizer^a^, *n* (%)	281 (8.1)	34 (4.6)	59 (8.9)	35 (6.2)	47 (9.2)	23 (9.8)	21 (7.8)	32 (12.7)	30 (12.5)
2 Mood stabilizers^a^	15 (0.4)	0 (0.0)	3 (0.5)	1 (0.2)	1 (0.2)	3 (1.3)	1 (0.4)	3 (1.2)	3 (0.1)
3 Mood stabilizers^a^	2 (0.1)	0 (0.0)	0 (0.0)	0 (0.0)	1 (0.2)	1 (0.4)	0 (0.0)	0 (0.0)	0 (0.0)
>3 Mood stabilizers^a^	1 (0.1)	0 (0.0)	0 (0.0)	0 (0.0)	0 (0.0)	0 (0.0)	1 (0.4)	0 (0.0)	0 (0.0)
Antiepileptics^b^, *n* (%)	138 (4.0)	17 (2.3)	0 (0.0)	22 (3.9)	21 (4.1)	16 (6.8)	16 (5.9)	26 (10.8)	20 (8.3)
Carbamazepine	6 (0.2)	0 (0.0)	0 (0)	0 (0.0)	0 (0.0)	0 (0.0)	0 (0.0)	0 (0.0)	6 (2.5)
Gabapentin	9 (0.3)	0 (0.0)	2 (0.3)	2 (0.4)	1 (0.2)	0 (0.0)	1 (0.4)	1 (0.4)	2 (0.8)
Lamotrigen	56 (1.6)	5 (0.7)	10 (1.5)	6 (1.1)	9 (1.8)	3 (1.3)	3 (1.1)	13 (5.4)	7 (2.9)
Pregabalin	113 (3.2)	8 (1.1)	44 (6.6)	8 (1.4)	11 (2.1)	12 (5.1)	13 (4.8)	12 (5)	5 (2.1)
Topiramate	4 (0.1)	0 (0.0)	3 (0.5)	0 (0.0)	0 (0.0)	0 (0.0)	1 (0.4)	0 (0.0)	0 (0.0)
Valproate	9 (0.3)	3 (0.4)	3 (0.5)	2 (0.4)	0 (0.0)	0 (0.0)	0 (0.0)	0 (0.0)	1 (0.4)
Other	19 (0.5)	1 (0.1)	2 (0.3)	5 (0.9)	1 (0.2)	3 (1.3)	1 (0.4)	5 (2.1)	1 (0.4)
Leveriracetam	7 (0.2)	0 (0.0)	0 (0.0)	0 (0.0)	0 (0.0)	2 (0.9)	1 (0.4)	3 (1.3)	1 (0.4)
Lacosamite	4 (0.1)	1 (0.1)	0 (0.0)	0 (0.0)	0 (0.0)	1 (0.4)	0 (0.0)	2 (0.8)	0 (0.0)
Ergenyl	1 (0.1)	0 (0.0)	0 (0.0)	1 (0.2)	0 (0.0)	0 (0.0)	0 (0.0)	0 (0.0)	0 (0.0)
Clonazepam	2 (0.1)	0 (0.0)	0 (0.0)	2 (0.4)	0 (0.0)	0 (0.0)	0 (0.0)	0 (0.0)	0 (0.0)
Chlorprotixen	1 (0.1)	0 (0.0)	0 (0.0)	0 (0.0)	1 (0.2)	0 (0.0)	0 (0.0)	0 (0.0)	0 (0.0)
Prothipendyl	1 (0.1)	0 (0.0)	0 (0.0)	1 (0.2)	0 (0.0)	0 (0.0)	0 (0.0)	0 (0.0)	0 (0.0)
Brivaracetam	1 (0.1)	0 (0.0)	0 (0.0)	1 (0.2)	0 (0.0)	0 (0.0)	0 (0.0)	0 (0.0)	0 (0.0)
No information	2 (0.1)	0 (0.0)	2 (0.3)	0 (0.0)	0 (0.0)	0 (0.0)	0 (0.0)	0 (0.0)	0 (0.0)
Lithium^b^, *n* (%)	132 (3.8)	17 (2.3)	23 (3.5)	14 (2.5)	30 (5.8)	15 (6.4)	7 (2.6)	12 (5.0)	14 (5.8)
Antipsychotics^b^, *n* (%)	874 (25.1)	159 (21.5)	154 (23.2)	147 (25.9)	166 (32.4)	61 (26.1)	54 (20.1)	65 (25.9)	68 (28.3)
Mean (SD)^a^	0.3 (0.5)	0.2 (0.5)	0.4 (0.6)	0.3 (0.5)	0.4 (0.6)	0.2 (0.4)	0.2 (0.5)	0.3 (0.5)	0.3 (0.5)
1 Antipsychotic^a^, *n* (%)	748 (21.5)	143 (19.4)	119 (17.9)	119 (21.0)	139 (27.1)	57 (24.4)	48 (17.8)	60 (23.9)	63 (26.3)
2 Antipsychotics^a^	117 (3.4)	16 (2.2)	29 (4.4)	28 (4.9)	26 (5.1)	4 (1.7)	5 (1.9)	5 (2.0)	4 (1.7)
3 Antipsychotics^a^	9 (0.3)	0 (0.0)	6 (0.9)	0 (0.0)	1 (0.2)	0 (0.0)	1 (0.4)	0 (0.0)	1 (0.4)
>3 Antipsychotics^a^	0 (0.0)	0 (0.0)	0 (0)	0 (0.0)	0 (0.0)	0 (0.0)	0 (0)	0 (0.0)	0 (0.0)
1^st^-Generation antipsychotics^b^, *n* (%)	185 (5.3)	18 (2.4)	88 (13.3)	39 (6.9)	8 (1.6)	4 (1.7)	11 (4.1)	10 (4.0)	7 (2.9)
Haloperidol	6 (0.2)	1 (0.1)	0 (0.0)	1 (0.2)	2 (0.4)	2 (0.9)	0 (0.0)	0 (0.0)	0 (0.0)
Other	185 (5.3)	36 (4.9)	88 (13.3)	38 (6.7)	6 (1.2)	1 (0.4)	11 (4.1)	5 (2.0)	0 (0.0)
Promethazine	39 (1.1)	9 (1.2)	–	19 (3.4)	2 (0.4)	0 (0.0)	0 (0.0)	2 (0.8)	7 (2.9)
Pipamperone	41 (1.2)	17 (2.3)	–	9 (1.6)	2 (0.4)	1 (0.4)	7 (2.6)	1 (0.4)	4 (1.7)
Prothipendyl	9 (0.3)	0 (0.0)	–	7 (1.2)	0 (0.0)	0 (0.0)	2 (0.7)	0 (0.0)	0 (0.0)
Melperone	13 (0.4)	8 (1.1)	–	0 (0.0)	2 (0.4)	0 (0.0)	2 (0.7)	1 (0.4)	0 (0.0)
Levomepromazin	3 (0.1)	0 (0.0)	–	1 (0.2)	0 (0.0)	0 (0.0)	0 (0.0)	0 (0.0)	2 (0.8)
Chlorprothexine	2 (0.1)	0 (0.0)	–	2 (0.4)	0 (0.0)	0 (0.0)	0 (0.0)	0 (0.0)	0 (0.0)
Promethazin	1 (0.1)	1 (0.1)	–	0 (0.0)	0 (0.0)	0 (0.0)	0 (0.0)	0 (0.0)	0 (0.0)
Melneurin	1 (0.1)	0 (0.0)	–	0 (0.0)	0 (0.0)	0 (0.0)	0 (0.0)	0 (0.0)	1 (0.4)
Fluspiriliene	1 (0.1)	1 (0.1)	–	0 (0.0)	0 (0.0)	0 (0.0)	0 (0.0)	0 (0.0)	0 (0.0)
Perazine	1 (0.1)	0 (0.0)	–	0 (0.0)	0 (0.0)	0 (0.0)	0 (0.0)	1 (0.4)	0 (0.0)
No information	88 (2.5)	0 (0.0)	88 (13.3)	0 (0.0)	0 (0.0)	0 (0.0)	0 (0.0)	0 (0.0)	0 (0.0)
2^nd^-Generation antipsychotics^a^, *n* (%)	660 (19.0)	144 (19.5)	0 (0.0)	123 (21.7)	161 (31.4)	59 (25.2)	47 (17.3)	63 (26.3)	63 (26.3)
Amisulpride	18 (0.5)	2 (0.3)	8 (1.2)	2 (0.4)	4 (0.8)	0 (0.0)	1 (0.4)	0 (0.0)	1 (0.4)
Aripiprazole	122 (3.5)	13 (1.8)	25 (3.8)	18 (3.2)	30 (5.8)	8 (3.4)	13 (4.8)	13 (5.4)	2 (0.8)
Cariprazine	4 (0.1)	0 (0.0)	2 (0.3)	0 (0.0)	2 (0.4)	0 (0.0)	0 (0.0)	0 (0.0)	0 (0.0)
Clozapine	8 (0.2)	3 (0.4)	2 (0.3)	0 (0.0)	0 (0.0)	1 (0.4)	0 (0.0)	0 (0.0)	2 (0.8)
Olanzapine	108 (3.1)	21 (2.8)	18 (2.7)	7 (1.2)	17 (3.3)	8 (3.4)	6 (2.2)	19 (7.9)	12 (5.0)
Quetiapine	476 (13.7)	78 (10.6)	70 (10.5)	87 (15.3)	93 (18.1)	38 (16.2)	30 (11.1)	32 (13.3)	48 (20.0)
Risperidone	97 (2.8)	21 (2.8)	14 (2.1)	16 (2.8)	36 (7)	5 (2.1)	1 (0.4)	1 (0.4)	3 (1.3)
Risperidone Depo	1 (0.1)	0 (0.0)	0 (0.0)	1 (0.2)	0 (0.0)	0 (0.0)	0 (0.0)	0 (0.0)	0 (0.0)
Ziprasidone	1 (0.1)	1 (0.1)	0 (0.0)	0 (0.0)	0 (0.0)	0 (0.0)	0 (0.0)	0 (0.0)	0 (0.0)
Other	23 (0.7)	2 (0.3)	12 (1.8)	4 (0.7)	4 (0.8)	1 (0.4)	0 (0.0)	0 (0.0)	0 (0.0)
Zotepine	1 (0.1)	0 (0.0)	–	0 (0.0)	0 (0.0)	1 (0.4)	0 (0.0)	0 (0.0)	0 (0.0)
Fluanxol	1 (0.1)	0 (0.0)	–	0 (0.0)	1 (0.2)	0 (0.0)	0 (0.0)	0 (0.0)	0 (0.0)
Levomepromazin	1 (0.1)	0 (0.0)	–	0 (0.0)	1 (0.2)	0 (0.0)	0 (0.0)	0 (0.0)	0 (0.0)
Melperon	6 (0.2)	2 (0.3)	–	4 (0.7)	0 (0.0)	0 (0.0)	0 (0.0)	0 (0.0)	0 (0.0)
Perazin	1 (0.1)	0 (0.0)	–	0 (0.0)	1 (0.2)	0 (0.0)	0 (0.0)	0 (0.0)	0 (0.0)
No information	13 (0.4)	0 (0.0)	12 (1.8)	0 (0.0)	1 (0.2)	0 (0.0)	0 (0.0)	0 (0.0)	0 (0.0)
Anxiolytics/hypnotics, *n* (%)	412 (11.8)	51 (6.9)	104 (15.7)	37 (6.5)	72 (14.0)	10 (4.3)	35 (3.0)	39 (16.3)	64 (26.7)
Mean (SD)^a^	0.1 (0.3)	0.1 (0.2)	0.1 (0.4)	0.1 (0.1)	0.1 (0.3)	0.1 (0.2)	0.1 (0.3)	0.2 (0.4)	0.3 (0.4)
Other psychiatric medication, *n* (%)	153 (4.4)	38 (5.1)	32 (4.8)	20 (3.5)	15 (2.9)	24 (10.3)	5 (1.9)	6 (2.5)	13 (5.4)

*Note*: Charité–CBF, Charité Campus Benjamin Franklin, Department of Psychiatry and Psychotherapy; Charité–Mitte, Charité Campus Mitte, Department of Psychiatry and Psychotherapy; Charité–St. Hedwig, St. Hedwig Hospital, Department of Psychiatry and Psychotherapy; KGU, Department of Psychiatry, Psychosomatic Medicine and Psychotherapy at the University Hospital Frankfurt; LMU, Clinic for Psychiatry and Psychotherapy at the Ludwig-Maximilian-University Munich; UKD, Department of Psychiatry and Psychotherapy at the University Hospital Carl Gustav Carus in Dresden; Uni Köln, Department of Psychiatry and Psychotherapy at the University Hospital of Cologne; ZI Mannheim, Central Institute of Mental Health, Department of Psychiatry and Psychotherapy in Mannheim.

Abbreviations: IQR, interquartile range; SD, standard deviation.

^a^Data from the Uni Köln is only available starting from patient 114 in the clinic.

^b^The sum of patients on individual medications can be larger than the number of patients per medication class due to co-treatment with more than one agent.

## Data Availability

The authors elect to not share data.

## Supporting Information

Supporting Information Table S1. F-diagnoses of comorbid psychiatric disorders. Table S2. Other comorbid disorders.
