# Supplementary material for: Clinical and Treatment Characteristics of 3795 Adults Consecutively Hospitalized for Major Depressive Disorder in the OASIS-D Study
Source: Depress Anxiety. 2025 Nov 26;2025:4470169. doi: 10.1155/da/4470169 (PMC12674865; doi:10.1155/da/4470169)
Supplement: Supporting Information — Table S1. F-diagnoses of comorbid psychiatric disorders. Table S2. Other comorbid disorders. [file 4470169.f1.pdf]

Supplementary Table 1: F-diagnoses of comorbid psychiatric disorders

**Comorbid psychiatric disorders**	**F-Diagnoses**
Dementia	F00, F01, F02, F03
Delirium due to known physiological condition	F05
Psychotic disorder with delusions due to known physiological condition	F06.2
Mood disorder due to known physiological condition	F06.3
Mild Cognitive Disorder	F06.7
Unspecified mental disorder due to brain damage and dysfunction and to physical disease	F06.9
Personality and behavioral disorders due to known physiological condition	F07
Alcohol use disorder	F10
Opioid use disorder	F11
Cannabinoid use disorder	F12
Sedatives/ hypnotics use disorder	F13
Cocaine use disorder	F14
Stimulants use disorder	F15
Hallucinogens use disorder	F16
Dysthymia	F34.1
Other recurrent mood [affective] disorders	F38.1
Phobic disorders and other anxiety disorders	F40, F41
Agoraphobia / social phobia/ specific phobia	F40.0, F40.00, F40.1, F40.2, F40.8, F40.9
Panic disorder (incl. agoraphobic with panic disorder)	F40.01, F41.0,
Generalized anxiety disorder	F41.1
Mixed anxiety-depressive disorder	F41.2
Anxiety, not otherwise specified	F41.8, F41.9
Obsessive-Compulsive disorder	F42
Reaction to severe stress and adjustment disorder	F43
Acute stress disorder	F43.0
Post-traumatic stress disorder	F43.1
Adjustment disorder	F43.2
Other reaction to severe stress	F43.8, F43.9
Dissociative disorder	F44
Somatoform disorder	F45
Neurasthenia	F48.0
Depersonalization-Derealization Syndrome	F48.1
Eating disorder	F50
Insomnia	F51
Sexual dysfunction not due to a substance or known physiological condition	F52
Mental and behavioral disorders associated with the puerperium	F53
Psychological and behavioral factors associated with disorders or diseases classified elsewhere	F54
Abuse of non-dependence-producing substances	F55
Personality disorder	F61, F62
Paranoid personality disorder	F60.0
Schizoid personality disorder	F21, F22, F60.1
Dissocial personality disorder	F60.2
Emotionally unstable personality disorder: impulsive type	F60.30
Emotionally unstable personality disorder, borderline type	F60.31
Histrionic personality disorder	F60.4
Obsessive-Compulsive (Anankastic) Personality Disorder	F60.5
Anxious (avoidant) personality disorder	F60.6
Other specific personality disorder/ Personality disorder, not otherwise specified	F60.8, F60.9
Mixed and other personality disorders	F61
Abnormal habits and impulse control disorders	F63
Gender Incongruence	F64
Disorders of sexual preference	F65
Other specified disorders of adult personality and behavior	F68.8
Unspecified disorder of adult personality and behavior	F69
Profound developmental disorder & intelligence disorder	F7, F84
Specific developmental disorders of speech and language	F80
Specific developmental disorders of scholastic skills	F81
Attention deficit and/ or hyperactivity disorder	F90, F98.80
Selective Mutism	F94.0
Tic disorder	F95

Supplementary Table 2: Other comorbid disorders

**Characteristic**	**Overall (n=3,795)**	**Charité - St. Hedwig (n=817)**	**Uni Köln (n=665)**	**KGU (n=607)**	**LMU (n=526)**	**Charité – Mitte (n=405)**	**ZI Mannheim (n=270)**	**Charité - CBF (n=254)**	**UKD (n=251)**
**Comorbid diagnoses, n (%)**	n=3,718	n=802	n=664	n=588	n=492	n=409	n=256	n=255	n=252
Profound developmental disorder and intelligence disorder	28 (0.8)	4 (0.5)	6 (0.9)	5 (0.8)	3 (0.6)	6 (1.5)	1 (0.4)	1 (0.4)	2 (0.8)
Abnormal habits and impulse control disorders	24 (0.6)	7 (0.9)	1 (0.1)	11 (1.9)	0 (0.0)	1 (0.2)	0 (0.0)	0 (0.0)	4 (1.6)
Dissociative disorder	23 (0.6)	7 (0.9)	1 (0.1)	4 (0.7)	0 (0.0)	1 (0.2)	2 (0.8)	2 (0.8)	6 (2.4)
Insomnia	14 (0.4)	1 (0.1)	1 (0.1)	1 (0.2)	1 (0.2)	2 (0.5)	3 (1.2)	3 (1.2)	2 (0.8)
Gender Incongruence	10 (0.3)	0 (0.0)	0 (0.0)	3 (0.5)	3 (0.6)	0 (0.0)	0 (0.0)	2 (0.8)	2 (0.8)
Dementia	7 (0.2)	3 (0.4)	4 (0.6)	0 (0.0)	0 (0.0)	0 (0.0)	0 (0.0)	0 (0.0)	0 (0.0)
Mild Cognitive Disorder	7 (0.2)	0 (0)	2 (0.3)	1 (0.2)	0 (0)	2 (0.5)	1 (0.4)	0 (0)	1 (0.4)
Depersonalization-Derealization Syndrome	4 (0.1)	0 (0.0)	1 (0.1)	3 (0.5)	0 (0.0)	0 (0.0)	0 (0.0)	0 (0.0)	0 (0.0)
Delirium due to known physiological condition	4 (0.1)	1 (0.1)	1 (0.1)	1 (0.2)	0 (0.0)	0 (0.0)	0 (0.0)	0 (0.0)	1 (0.4)
Tic disorder	3 (0.1)	0 (0.0)	0 (0.0)	2 (0.3)	0 (0.0)	1 (0.2)	0 (0.0)	0 (0.0)	0 (0.0)
Sexual dysfunction not due to a substance or known physiological condition	3 (0.1)	0 (0.0)	0 (0.0)	1 (0.2)	0 (0.0)	1 (0.2)	1 (0.4)	0 (0.0)	0 (0.0)
Mental and behavioral disorders associated with the puerperium	3 (0.1)	2 (0.2)	1 (0.1)	0 (0.0)	0 (0.0)	0 (0.0)	0 (0.0)	0 (0.0)	0 (0.0)
Disorders of sexual preference	2 (0.1)	0 (0.0)	0 (0.0)	0 (0.0)	1 (0.2)	0 (0.0)	0 (0.0)	1 (0.4)	0 (0.0)
Selective Mutism	2 (0.1)	0 (0.0)	0 (0.0)	0 (0.0)	0 (0.0)	1 (0.2)	0 (0.0)	0 (0.0)	1 (0.4)
Psychotic disorder with delusions due to known physiological condition	2 (0.1)	0 (0.0)	0 (0.0)	1 (0.2)	0 (0.0)	1 (0.2)	0 (0.0)	0 (0.0)	0 (0.0)
Personality and behavioral disorders due to known physiological condition	2 (0.1)	0 (0.0)	0 (0.0)	0 (0.0)	0 (0.0)	2 (0.5)	0 (0.0)	0 (0.0)	0 (0.0)
Psychological and behavioral factors associated with disorders or diseases classified elsewhere	1 (0.0)	0 (0.0)	0 (0.0)	0 (0.0)	0 (0.0)	0 (0.0)	1 (0.4)	0 (0.0)	0 (0.0)
Abuse of non-dependence-producing substances	1 (0.0)	1 (0.1)	0 (0.0)	0 (0.0)	0 (0.0)	0 (0.0)	0 (0.0)	0 (0.0)	0 (0.0)
Specific developmental disorders of speech and language	1 (0.0)	1 (0.1)	0 (0.0)	0 (0.0)	0 (0.0)	0 (0.0)	0 (0.0)	0 (0.0)	0 (0.0)
Specific developmental disorders of scholastic skills	1 (0.0)	0 (0.0)	0 (0.0)	0 (0.0)	0 (0.0)	0 (0.0)	1 (0.4)	0 (0.0)	0 (0.0)
Other specified disorders of adult personality and behavior	1 (0.0)	0 (0.0)	0 (0.0)	1 (0.2)	0 (0.0)	0 (0.0)	0 (0.0)	0 (0.0)	0 (0.0)
Mood disorder due to known physiological condition	1 (0.0)	0 (0.0)	0 (0.0)	1 (0.2)	0 (0.0)	0 (0.0)	0 (0.0)	0 (0.0)	0 (0.0)
Neurasthenia	1 (0.0)	0 (0.0)	0 (0.0)	0 (0.0)	1 (0.2)	0 (0.0)	0 (0.0)	0 (0.0)	0 (0.0)

Characteristic	Overall (n=3,795)	Charité - St. Hedwig (n=817)	Uni Köln (n=665)	KGU (n=607)	LMU (n=526)	Charité – Mitte (n=405)	ZI Mannheim (n=270)	Charité - CBF (n=254)	UKD (n=251)
Unspecified mental disorder due to brain damage, dysfunction, and physical disease	1 (0.0)	0 (0.0)	0 (0.0)	0 (0.0)	0 (0.0)	1 (0.2)	0 (0.0)	0 (0.0)	0 (0.0)
Other recurrent mood [affective] disorders	1 (0.0)	0 (0.0)	0 (0.0)	0 (0.0)	0 (0.0)	0 (0.0)	0 (0.0)	1 (0.4)	0 (0.0)
Unspecified disorder of adult personality and behavior	1 (0.0)	0 (0.0)	0 (0.0)	0 (0.0)	0 (0.0)	0 (0.0)	0 (0.0)	0 (0.0)	1 (0.4)

Abbreviation: Charité – CBF: Charité Campus Benjamin Franklin, Department of Psychiatry and Psychotherapy; Charité – Mitte: Charité Campus Mitte, Department of Psychiatry and Psychotherapy; Charité - St. Hedwig: St. Hedwig Hospital, Department of Psychiatry and Psychotherapy; IQR: interquartile range; KGU: Department of Psychiatry, Psychosomatic Medicine and Psychotherapy at the University Hospital Frankfurt; LMU: Clinic for Psychiatry and Psychotherapy at the Ludwig-Maximilian-University Munich; UKD: Department of Psychiatry and Psychotherapy at the University Hospital Carl Gustav Carus in Dresden; Uni Köln: Department of Psychiatry and Psychotherapy at the University Hospital of Cologne; ZI Mannheim: Central Institute of Mental Health, Department of Psychiatry and Psychotherapy in Mannheim
